# Dexmedetomidine Protects Human Cardiomyocytes Against Ischemia-Reperfusion Injury Through α2-Adrenergic Receptor/AMPK-Dependent Autophagy

**DOI:** 10.3389/fphar.2021.615424

**Published:** 2021-05-21

**Authors:** Yingying Xiao, Junpeng Li, Lisheng Qiu, Chuan Jiang, Yanhui Huang, Jinfen Liu, Qi Sun, Haifa Hong, Lincai Ye

**Affiliations:** ^1^ Department of Thoracic and Cardiovascular Surgery, Shanghai Children’s Medical Center, School of Medicine, Shanghai Jiaotong University, Shanghai, China; ^2^ Hwa Mei Hospital, University of Chinese Academy of Sciences, Ningbo, China; ^3^ Shanghai Institute for Pediatric Congenital Heart Disease, Shanghai Children’s Medical Center, School of Medicine, Shanghai Jiaotong University, Shanghai, China; ^4^ Institute of Pediatric Translational Medicine, Shanghai Children’s Medical Center, School of Medicine, Shanghai Jiaotong University, Shanghai, China; ^5^ Department of Anesthesiology, Shanghai Children’s Medical Center, School of Medicine, Shanghai Jiaotong University, Shanghai, China

**Keywords:** dexmedetomidine, cardiomyocyte, ischemia-reperfusion injury, congenital heart disease, autophagy

## Abstract

**Background:** Ischemia-reperfusion injury (I/R) strongly affects the prognosis of children with complicated congenital heart diseases (CHDs) who undergo long-term cardiac surgical processes. Recently, the α2-adrenergic receptor agonist *Dexmedetomidine* (Dex) has been reported to protect cardiomyocytes (CMs) from I/R in cellular models and adult rodent models. However, whether and how Dex may protect human CMs in young children remains largely unknown.

**Methods and Results:** Human ventricular tissue from tetralogy of Fallot (TOF) patients and CMs derived from human-induced pluripotent stem cells (iPSC-CMs) were used to assess whether and how Dex protects human CMs from I/R. The results showed that when pretreated with Dex, the apoptosis marker-TUNEL and cleaved caspase 3 in the ventricular tissue were significantly reduced. In addition, the autophagy marker LC3II was significantly increased compared with that of the control group. When exposed to the hypoxia/reoxygenation process, iPSC-CMs pretreated with Dex also showed reduced TUNEL and cleaved caspase 3 and increased LC3II. When the autophagy inhibitor (3-methyladenine, 3-MA) was applied to the iPSC-CMs, the protective effect of Dex on the CMs was largely blocked. In addition, when the fusion of autophagosomes with lysosomes was blocked by Bafilomycin A1, the degradation of p62 induced by Dex during the autophagy process was suspended. Moreover, when pretreated with Dex, both the human ventricle and the iPSC-CMs expressed more AMP-activated protein kinase (AMPK) and phospho AMPK (pAMPK) during the I/R process. After AMPK knockout or the use of an α2-adrenergic receptor antagonist-yohimbine, the protection of Dex and its enhancement of autophagy were inhibited.

**Conclusion:** Dex protects young human CMs from I/R injury, and α2-adrenergic receptor/AMPK-dependent autophagy plays an important role during this process. Dex may have a therapeutic effect for children with CHD who undergo long-term cardiac surgical processes.

## Introduction

Normal heart function requires sufficient blood flow to carry the oxygen and nutrients that support the electrophysiological activity of cardiomyocytes (CMs). Typically, surgeons must utilize cardiopulmonary bypass (CPB) to operate on children with complicated congenital heart diseases (CHDs). During CPB, CMs will experience ischemia. When the operation has concluded, CMs undergo a reperfusion process (Hascoet et al., 2020). The ischemia/ reperfusion (I/R) process can induce CM death, which is called I/R injury (Wang et al., 2020). CM damage and postoperative heart failure after surgery are the primary causes of postoperative death in complicated CHD cases (Nieminen et al., 2007; Spector et al., 2018; Wang et al., 2020). As such, there is a critical need to protect cardiomyocytes from I/R injury in cases of complicated CHDs (Nieminen et al., 2007; Spector et al., 2018; Wang et al., 2020).

Dexmedetomidine (Dex) is a highly selective α2-adrenergic receptor agonist that is primarily used for sedation and analgesia after anesthesia (Bailey, 2020). There is increasing evidence that Dex has protective effects on I/R injury for several important organs (Torregroza et al., 2020). Additionally, Dex has also been widely used in perioperative anesthesia maintenance for cardiac surgery in infants and young children (Dex 0.2–0.8 ug·kg^−1^·h^−1^) (Zimmerman et al., 2019) and has achieved good clinical results (Bush et al., 2018; Zhou et al., 2019; Zimmerman et al., 2019). Recently, several studies have also demonstrated that Dex is effective for I/R injury protection in the adult rodent heart (Du et al., 2019; Yuan et al., 2019; Chang et al., 2020). However, because the response of infant CMs to hypoxia and the stimulation of the surrounding environment are quite different from those of adult CMs (Sun et al., 2019; Ye et al., 2020), whether Dex is effective in I/R protection for human infant CM requires intensive investigation.

Autophagy is a critical process for the maintenance of intracellular homeostasis in CMs (Bravo-San Pedro et al., 2017). During autophagy, autophagosomes fuse with lysosomes to degrade the engulfed contents that include damaged proteins and cytoplasmic organelles (Laker et al., 2017). Autophagosome formation is regulated by unc-51, similar to autophagy activating kinase 1 (Ulk1). A previous report demonstrated that the AMP-activated protein kinase (AMPK) phosphorylation of Ulk1 was required for the mitochondrial autophagy process in skeletal muscle (Laker et al., 2017). Damaged mitochondria are a source of reactive oxygen species (ROS), which cause severe damage to CM function (Li et al., 2020; Pei et al., 2020). Our previous study indicated that Dex increased the expression of AMPK and reduced the ROS production in a mouse I/R model (Sun et al., 2017). Thus, in the current study, it is investigated whether Dex will enhance autophagy *via* the AMPK signaling pathway in human I/R samples and in CMs derived from human-induced pluripotent stem cells (iPSC-CMs).

The Simple Western^™^ system (Wes) uses capillary electrophoresis to identify and quantitate proteins of interest (only 3 μg protein required for one experiment), avoiding the protein separation and transference that occur when using the traditional Western blot method (Harris, 2015). Wes is becoming increasingly popular and has achieved good results (Bezzerides et al., 2019; Reyes-Serratos et al., 2020; Wiswell et al., 2020). Because human atria samples and iPSC-CMs are limited and valuable, Wes is used in this study to detect small amounts of proteins.

## Materials and Methods

All of the reagents and antibodies used in this study are detailed in Supplementaary Tables S1, S2.

### Human Sample Collection

Twelve right-ventricular-outflow myocardial tissue specimens were collected from resections required to relieve obstructions in tetralogy of Fallot (TOF) patients admitted to the Shanghai Children’s Medical Center, Shanghai, China between May 2020 and July 2020. Six were pretreated with Dex [0.8 ug·kg^−1^·h^−1^, a concentration in regular use in the hospital (Keating, 2015)] prior to CPB, while the other six were not treated with Dex. In addition to the Dex treatment, the other treatments were the same for both groups. Each specimen was quickly placed in ice-cold cardioplegia (KH_2_PO_4_ 50, MgSO_4_ 8, Adenosine 5, hepes 10, mannitol 100, taurine 10, and glucose 140 mM, pH 7.4) and transferred to a cell culture room. The tissues were then cultured with pre-oxygenated DMEM/F12 for 1 h using 100% O_2_ bubbling. Next, the tissues were washed and divided into two parts for immunostaining and Wes. All of the procedures conformed to the principles outlined in the Declaration of Helsinki and were approved by the Animal Welfare and Human Studies Committee at Shanghai Children’s Medical Center. Written informed consent was obtained from the parents of each patient prior to study initiation.

### Induced Pluripotent Stem Cell Differentiation, Maintenance, and O_2_ Treatment

The human-induced pluripotent stem cell (iPSC) line (del-AR1034ZIMA 00), derived from healthy male dermal fibroblasts, was purchased from Allele Biotechnology (Kadari et al., 2014 Apr 8). The iPSC cell line (HEBHMUi002-A) was stored in our lab, generated from peripheral blood mononuclear cells of a healthy 39-year-old female (Ma et al., 2020 Jan). The cells were differentiated under normal O_2_ (21%) conditions and maintained using the STEMdiff Cardiomyocyte Differentiation Kit according to the manufacturer’s instructions. After 15 days of induction, approximately 90% of the cells were beating and positive for both cardiac troponin T (cTnT) and sarcomeric α-actinin (SAA). The cells were reseeded and cultured in an environment using a 1% O_2_ concentration in incubators for 12 h, after which they were returned to 21% O_2_ for 24 h. To evaluate the effect of Dex, 5 uM Dex was added into the culture media 1 h before the media was transferred into a 1% O_2_ incubator, according to the methods found in previous publications (Liu et al., 2018; Li et al., 2019; Peng et al., 2020). To evaluate the effect of autophagy, the autophagy inhibitor, 3-MA (5 mM), was added into the culture media 2 h before it was transferred into a 1% O_2_ incubator. To evaluate the autophagy flux induced by Dex, the autophagosomal maturation inhibitor-bafilomycin A1 (BafA1, 1 μM) was added into the culture media 2 h before it was transferred into a 1% O_2_ incubator. To evaluate whether the autophagy flux induced by Dex was dependent on α2-adrenergic receptors, the α2-adrenergic receptor antagonist yohimbine (100 μM) was added into the culture media 2 h before it was transferred into a 1% O_2_ incubator. The cells were then subjected to Wes and immunofluorescence.

### siRNA transfection

The commercial human AMPK siRNA and scramble siRNA were purchased from Santa Cruz Biotechnology (Santa Cruz, CA, United States). The iPSC-CMs were separately plated on 24-well plates at 5 x 10^4^ cells per well in 2 ml of antibiotic-free normal growth medium supplemented with fetal bovine serum (FBS). The cells reached 60–70% confluence. They were transfected with scrambled siRNA or the AMPK siRNA duplex (100 pmol/L) using Lipofectamine 2000 (Invitrogen). The cells were harvested after 48 h of transfection for further experiments.

### Immunofluorescence

After fixation with 4% paraformaldehyde, the slides or cells were permeated with 0.5% Triton X-100 for 15 min, blocked using a 10% donkey serum for 30 min, and stained with a TdT-mediated dUTP Nick-End Labeling (TUNEL) Kit according to the manufacturer’s instructions. In brief, the slides or cells were incubated with the TUNEL cocktail for 1 h. After washing with phosphate-buffered saline (PBS) three times, the slides or cells were incubated with SAA antibodies overnight at 4°C. On the next day, the slides or cells were incubated with secondary antibodies and 4’,6-diamidino-2-phenylindole (DAPI) for 30 min. Three researchers, who were blind to the sample identities, quantified TUNEL by either manual counting or digital thresholding. This included image segmentation and the creation of a binary image from the grayscale. The converted binary images were analyzed using ImageJ software (NIH, Bethesda, MA, United States; Laboratory for Optical and Computational Instrumentation, University of Wisconsin, Madison, WI, United States).

### Capillary of the Western Blot Analysis

Proteins were extracted using the RIPA Lysis Buffer according to the manufacturer’s instructions (P0013B, Beyotime, Shanghai, China). In brief, the tissues were homogenized and extracted using the RIPA buffer for 5 min on ice, and centrifuged using 14,000 *g* for 5 min. The supernatant was then collected. The quantification of proteins was achieved using the Wes (ProteinSimple, CA, United States) according to the manufacturer’s instructions (Reyes-Serratos et al., 2020). In brief, protein (3 μg), primary antibodies, second antibodies, and the HRP conjunction were loaded into the Wes simple plates. The plates were loaded into the detection machine after centrifugation (2,500 *g* for 5 min).

### Statistical Analysis

Continuous data, including the mRNA expression, protein expression, and the number of TUNEL-positive cells, were expressed as the means ± standard deviations. The differences were evaluated using a Student’s *t*-test, ANOVA, or [the Student–Newman–Keuls (SNK) test for the post hoc tests] if the data were normally distributed. Otherwise, they were tested using the rank sum test. *p*-values <0.05 were considered to be statistically significant. The statistical analyses were performed using SAS software version 9.2 (SAS Institute Inc., Cary, NC, United States).

## Results

### Patient Clinical Information

Due to the patient age, the pressure load and SaO_2_ contribute to the oxidative DNA damage of CMs (Huang et al., 2017; Ye et al., 2020; Ye et al., 2020). Therefore, patients were selected to ensure that there were no significant differences in age, SaO_2_, or pulmonary arterial pressure (increasing the right ventricular pressure load) between the two groups (Table 1).

**TABLE 1:** Patient clinical information

Group	Sample number	Age(months)	Disease	Sex	SaO_2_(%)	PAH(mmHg)
I/R	1	18	TOF	Female	88	76.7
	2	17	TOF	Female	87	88
	3	21	TOF	Male	88	84
	4	18	TOF	Male	90	92
	5	15	TOF	Male	80	80
	6	24	TOF	Male	88	84
	Average	18.83	/	/	86.8	84.1
	SD	3.2	/	/	3.5	5.5
I/R+Dex	7	17	TOF	Male	80	96
	8	17	TOF	Male	88	73
	9	14	TOF	Male	86	88
	10	19	TOF	Male	81	102
	11	24	TOF	Female	84	88
	12	24	TOF	Female	87	86
—	Average	19.2	/	/	84.3	88.8
—	SD	4.1	/	/	3.3	9.9
P	—	0.8776	/	/	0.2289	0.3291

Dex protects human ventricular tissue from I/R injury and is associated with autophagy upregulation

**FIGURE 1:**
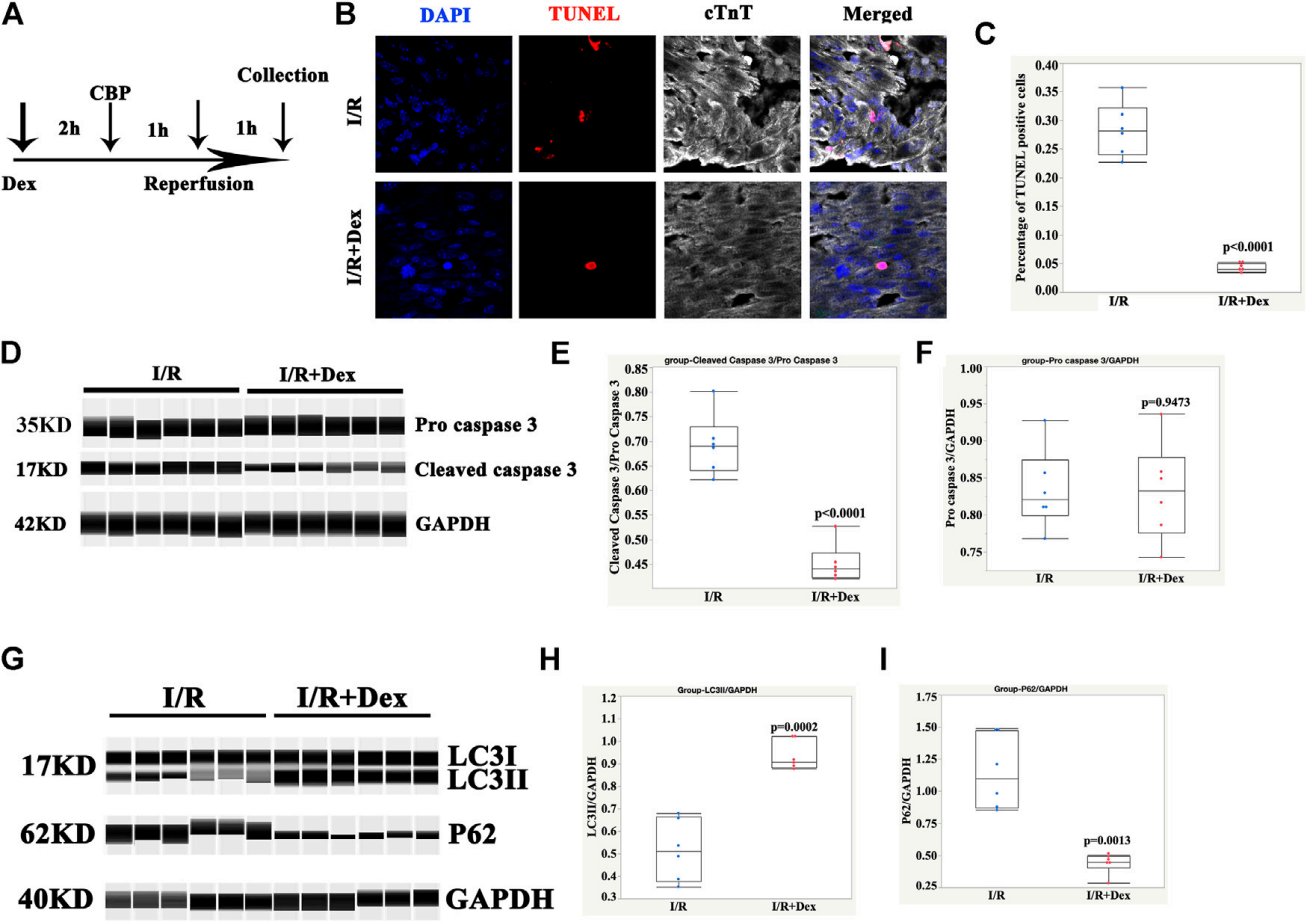
Dex protected human ventricular tissue from apoptosis and was associated with autophagy. **(A)** Timelines of the Dex, cardiopulmonary bypass (CBP), and reperfusion treatments. **(B)** Representative immunofluorescence images of the ventricular tissues pretreated with Dex and the control tissues. Blue (DAPI), red (TUNEL), and white (cTnT). **(C)** Quantification of the TUNEL positive cells. N = 6 patients, ten slides/patient. **(D)** Representative pro-cleaved caspase 3 Wes blot of atrial tissues pretreated with Dex. **(E)** Quantification of the cleaved caspase 3 relative expression. N = 6 patients. **(F)** Quantification of the pro-caspase 3 relative expression. N = 6 patients. **(G)** Representative LC3/p62 Wes blot of the atrial tissues pretreated with Dex. **(H)** Quantification of the LC3II relative expression. N = 6 patients. **(I)** Quantification of the p62 relative expression. N = 6 patients.

As shown in Figure 1A, the Dex pretreatment occurred 2 h before the CBP, the duration of the CBP was 1 h, and the reperfusion duration (exposure to the pre-oxygenated culture media) was 1 h. The immunofluorescence results showed that the number of TUNEL- positive cells was dramatically lower in the Dex-pretreated tissues than in the control group (Figures 1B,C). To further confirm the results, the Wes simple protein detection system, which can detect small amounts of protein, was applied. The results showed that the protein levels of cleaved caspase 3 in the Dex-pretreated group were significantly reduced compared with levels in the control group (Figures 1D–F). These results suggested that Dex protected the human ventricle from I/R injury.

The autophagy marker, LC3II, was significantly increased in the Dex-pretreated group compared with the control group (Figures 1G,H). Consistent with an increase in autophagy, p62/SQSTM1, a polyubiquitin-binding protein, was degraded during autophagy. In addition, it displayed a reverse change in LC3II (Figures 1G,I). These results indicated that the protection of Dex may be associated with autophagy.

Dex protected human iPSC-CMs from the hypoxia/reoxygenation (H/R) process and was associated with autophagy upregulation

**FIGURE 2:**
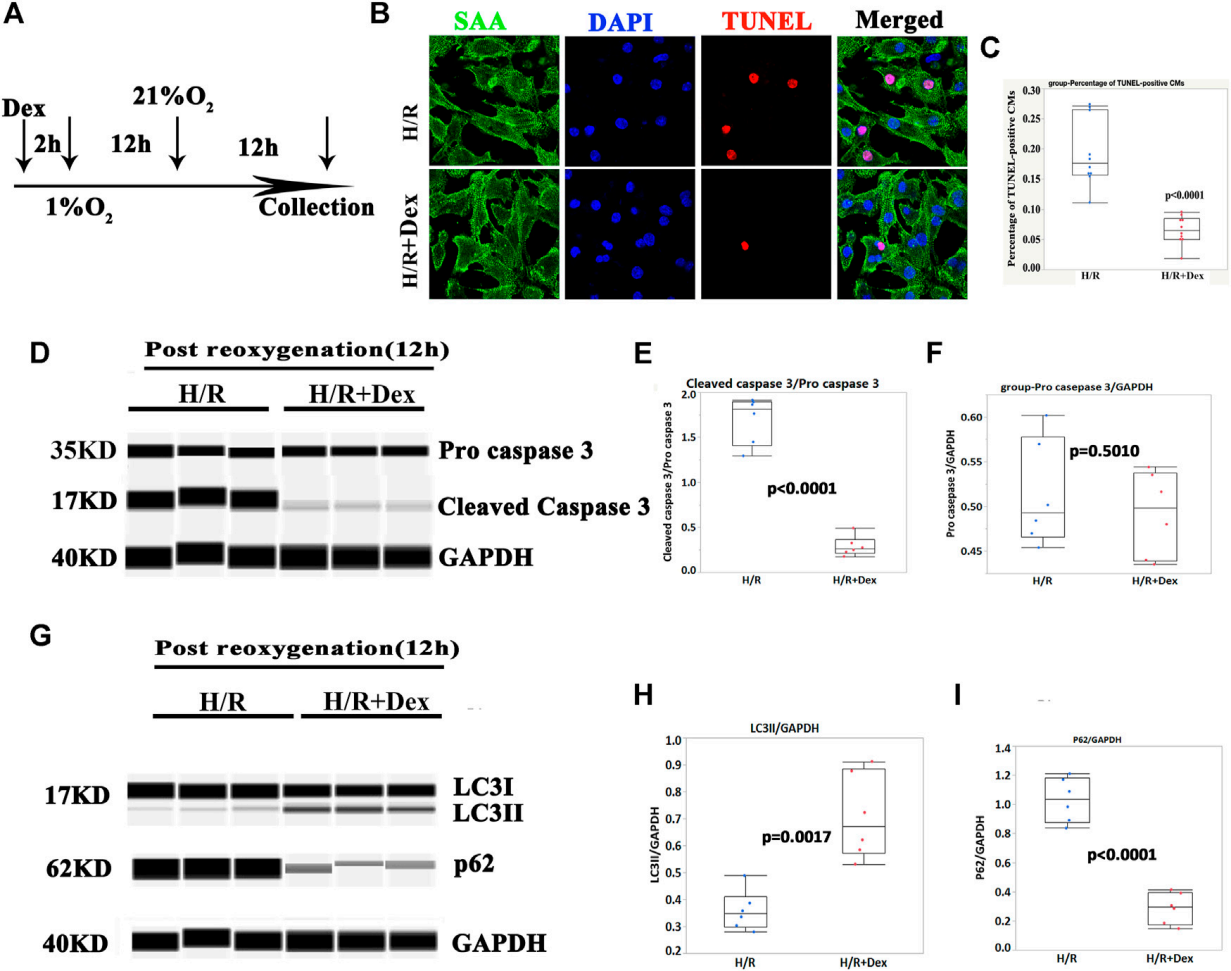
Dex protected human iPSC-CMs from apoptosis and was associated with autophagy. **(A)** Timelines of the Dex, hypoxia (1% O_2_) and reoxygenation (21% O_2_) treatments. **(B)** Representative immunofluorescence images of the iPSC-CM pretreated with Dex. Blue (DAPI), red (TUNEL), and green (sarcomeric α- actin, SAA). **(C)** Quantification of the TUNEL-positive cells. N= 10 fields from three independent experiments. **(D)** Representative pro-cleaved caspase 3 Wes blot of the iPSC-CMs pretreated with Dex at the time of post-reoxygenation (12 h). **(E)** Quantification of the cleaved caspase 3 relative expression. N = 6 replicates. **(F)** Quantification of the pro- caspase 3 relative expression. N = 6 replicates. **(G)** Representative LC3/p62 Wes blot of the iPSC-CMs pretreated with Dex at the time of post-reoxygenation (12 h). **(H)** Quantification of the LC3II relative expression. N = 6 replicates. **(I)** Quantification of the p62 relative expression. N = 6 replicates.

To confirm the *in vivo* results, human iPSC-CMs were used to confirm the protective effect of Dex *in vitro*. As shown in Figure 2A, Dex was pretreated prior to hypoxia (1% O_2_) for 2 h, the duration of hypoxia was 12 h, and the reoxygenation duration before CM collection was 12 h. The results demonstrated that Dex significantly reduced the number of TUNEL-positive CMs *in vitro* (Figures 2B,C; Supplementary Figures S1A,B), and the Wes results were consistent with the immunostaining results (Figures 2D–F; Supplementary Figures S1C–E). These results indicated that Dex protected human iPSC-CM from H/R injury *in vitro*.

The autophagy marker, LC3II, was significantly increased in the Dex-pretreatment group compared with the control group (Figures 2G,H; Supplementary Figures S1F,G), and p62/SQSTM1 showed a reverse change in LC3II (Figures 2G,I; Supplementary Figures S1F,H). These results indicated that the protection of Dex *in vitro* may be associated with autophagy.

The autophagy inhibitor, 3-MA, blocked the protective effect of Dex

**FIGURE 3:**
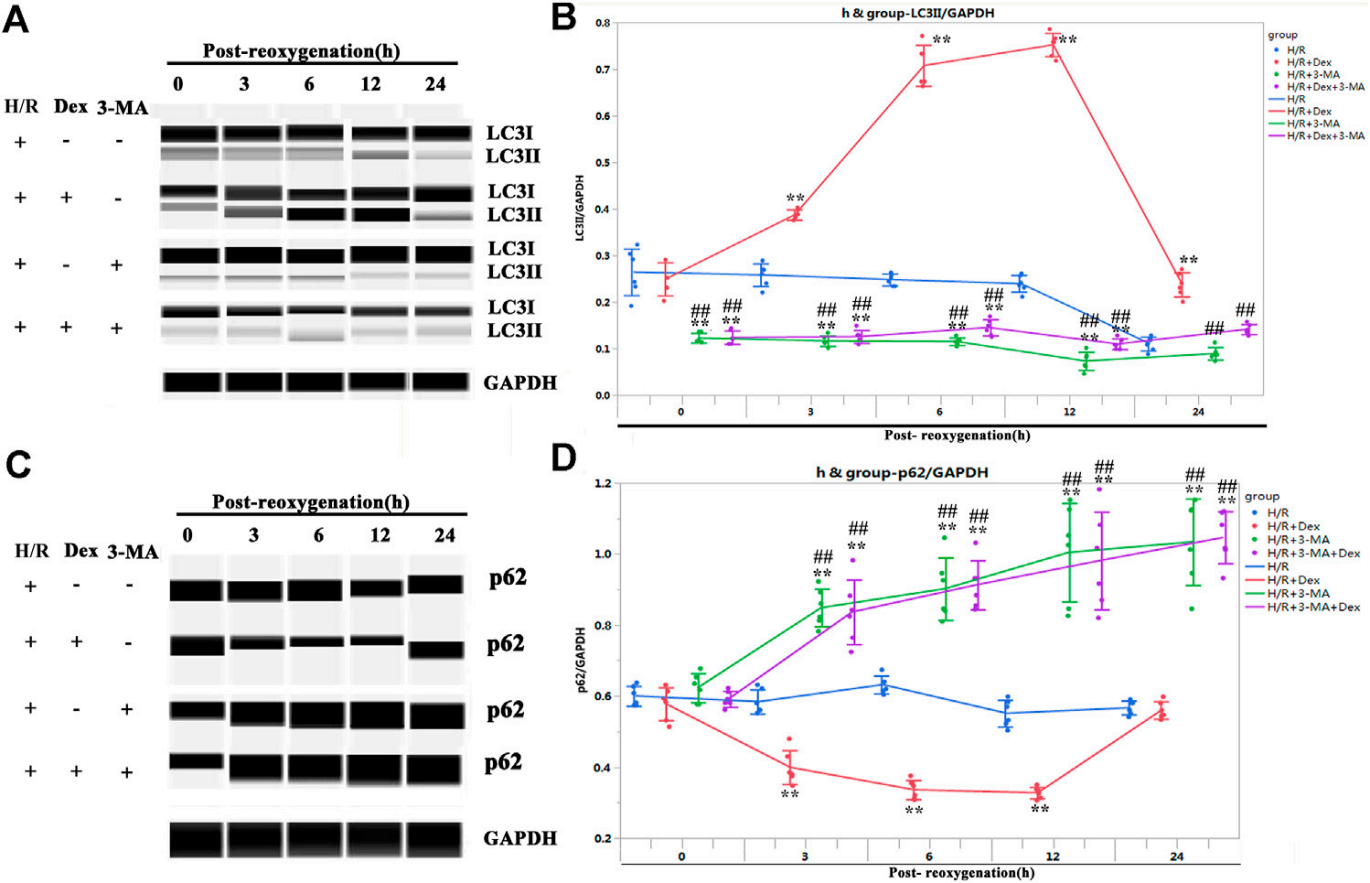
Autophagy inhibitor (3-methyladenine, 3-MA) blocked the autophagy flux induced by Dex. **(A)** Representative LC3II Wes blots of iPSC-CMs pretreated with Dex and 3-MA. **(B)** Quantification of the LC3II relative expression. N = 6 replicates. **(C)** Representative p62 Wes blots of iPSC-CMs pretreated with Dex and 3-MA. **(D)** Quantification of the p62 relative expression. N = 6 replicates. ** *p* <0.01. vs. H/R; ## *p* <0.01, vs. H/R + Dex.

**FIGURE 4:**
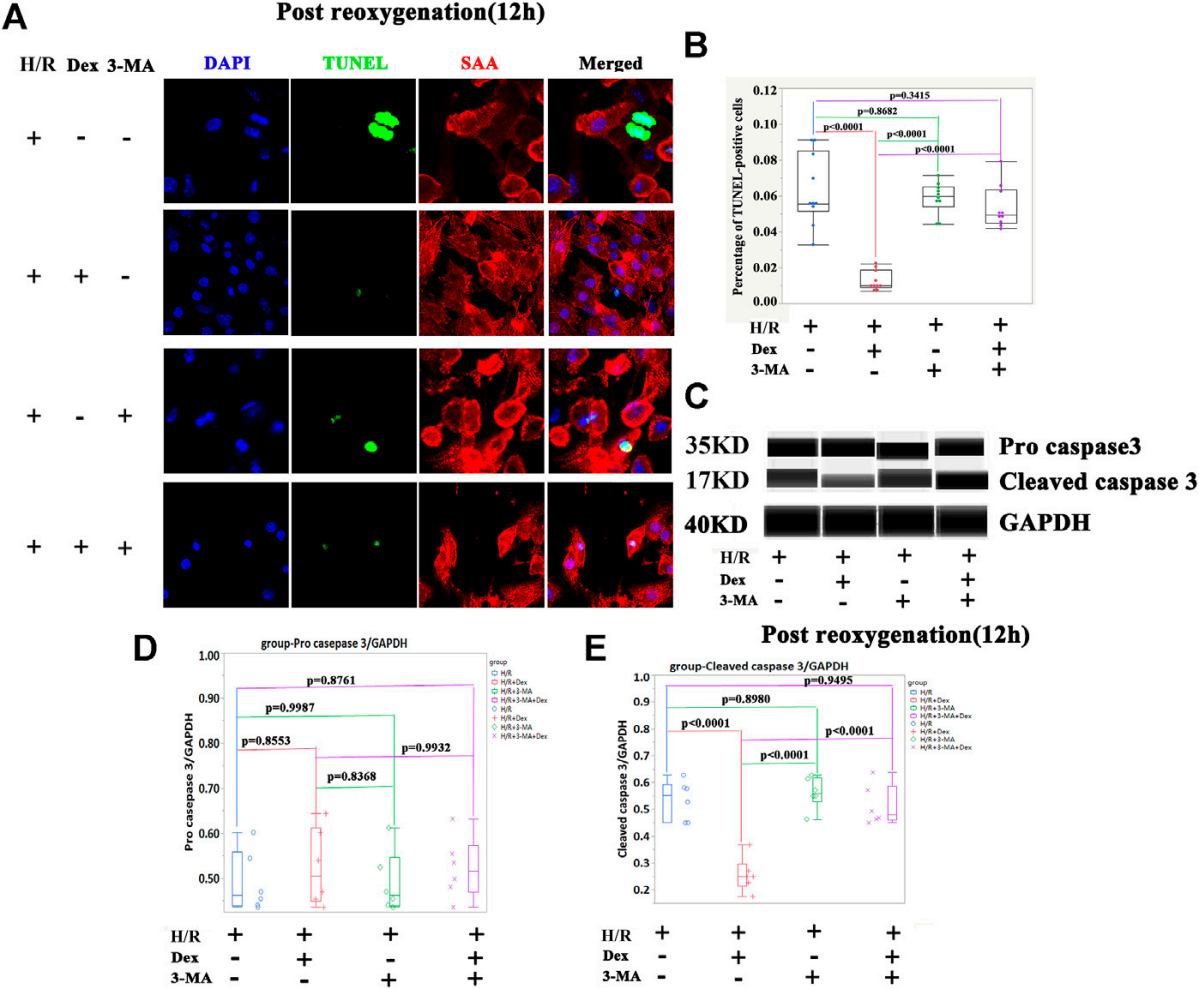
The autophagy inhibitor (3-methyladenine, 3-MA) blocked the protection of Dex during I/R injury. **(A)** Representative TUNEL immunofluorescence images of iPSC-CMs pretreated with 3-methyladenine (3-MA). Blue (DAPI), green (TUNEL), red (sarcometric α-actin, SAA). **(B)** Quantification of the TUNEL- positive cells. N= 10 fields from three independent experiments. **(C)** Representative pro-cleaved caspase 3 Wes blot of iPSC-CM pretreated with Dex and 3-MA. **(D)** Quantification of the pro-caspase 3 relative expression. N = 6 replicates. **(E)** Quantification of the cleaved caspase 3 relative expression. N= 6 replicates.

The results from the *in vivo* and *in vitro* studies indicated that autophagy may be associated with the protective effect of Dex. To verify the role of autophagy in Dex’s I/R protection, the autophagy inhibitor-3-MA, a PI3-Kinase (PI3K) inhibitor, was introduced (Petiot et al., 2000). The inhibition of PI3K impedes the recruitment of LC3I to the autophagosomal membrane (Petiot et al., 2000). The results demonstrated that the 3-MA significantly reduced the expression of LC3II at the beginning of the post-reoxygenation period (0 h) (Figures 3A,B). The downregulation of LC3II lasted for 12 h (Figures 3A,B). The increase in the LC3II expression by Dex was completely blocked by 3-MA (Figures 3A,B). Consistent with the LC3II expression, p62 showed a reverse pattern of expression (Figures 3C,D). As a result, at the time of post-oxygenation (12 h), the number of TUNEL-positive CMs and the expression of cleaved caspase 3 were significantly increased (Figures 4A–E). These results demonstrated that autophagy played a critical role in the protective effect of Dex in I/R.

### Dex Activated Autophagy but Did not Block Autophagosomal Maturation

**FIGURE 5:**
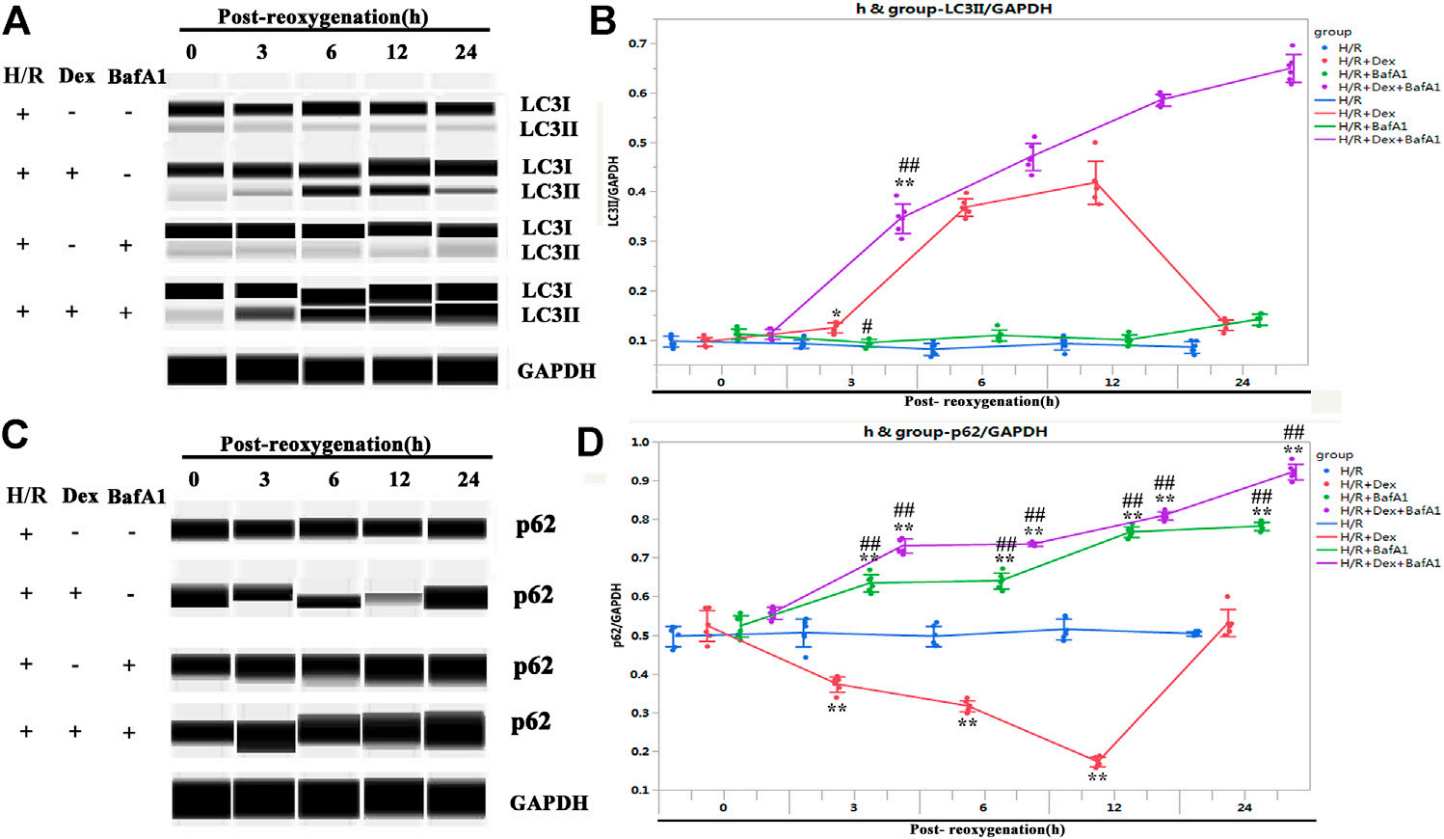
Dex activated autophagy but did not block autophagosomal maturation. **(A)** Representative LC3II Wes blot of iPSC-CM pretreated with Dex and BafA1 (the inhibitors of autophagosomal maturation). **(B)** Quantification of the LC3II relative expression. N= 6 replicates. **(C)** Representative p62 Wes blot of the iPSC-CM pretreated with Dex and BafA1. **(D)** Quantification of the p62 relative expression. N= 6 replicates. * *p* <0.05, ** *p* <0.01. vs. H/R; ## *p* <0.01, vs. H/R + Dex.

To confirm the upregulation of LC3II induced by the Dex treatment represented the activation of autophagy rather than a blockage in autophagosomal maturation, the iPSC-CMs were treated with Bafilomycin A1(BafA1), an inhibitor that blocks the fusion of autophagosomes with lysosomes (Bowman et al., 1988). As shown in Figures 5A–D, BafA1 caused significant accumulations of both LC3II and p62, indicating that the increase in LC3II induced by Dex did not occur because of a downstream inhibition in the autophagic flux.

### The Autophagy Induced by Dex was AMP-Activated Protein Kinase Dependent

**FIGURE 6:**
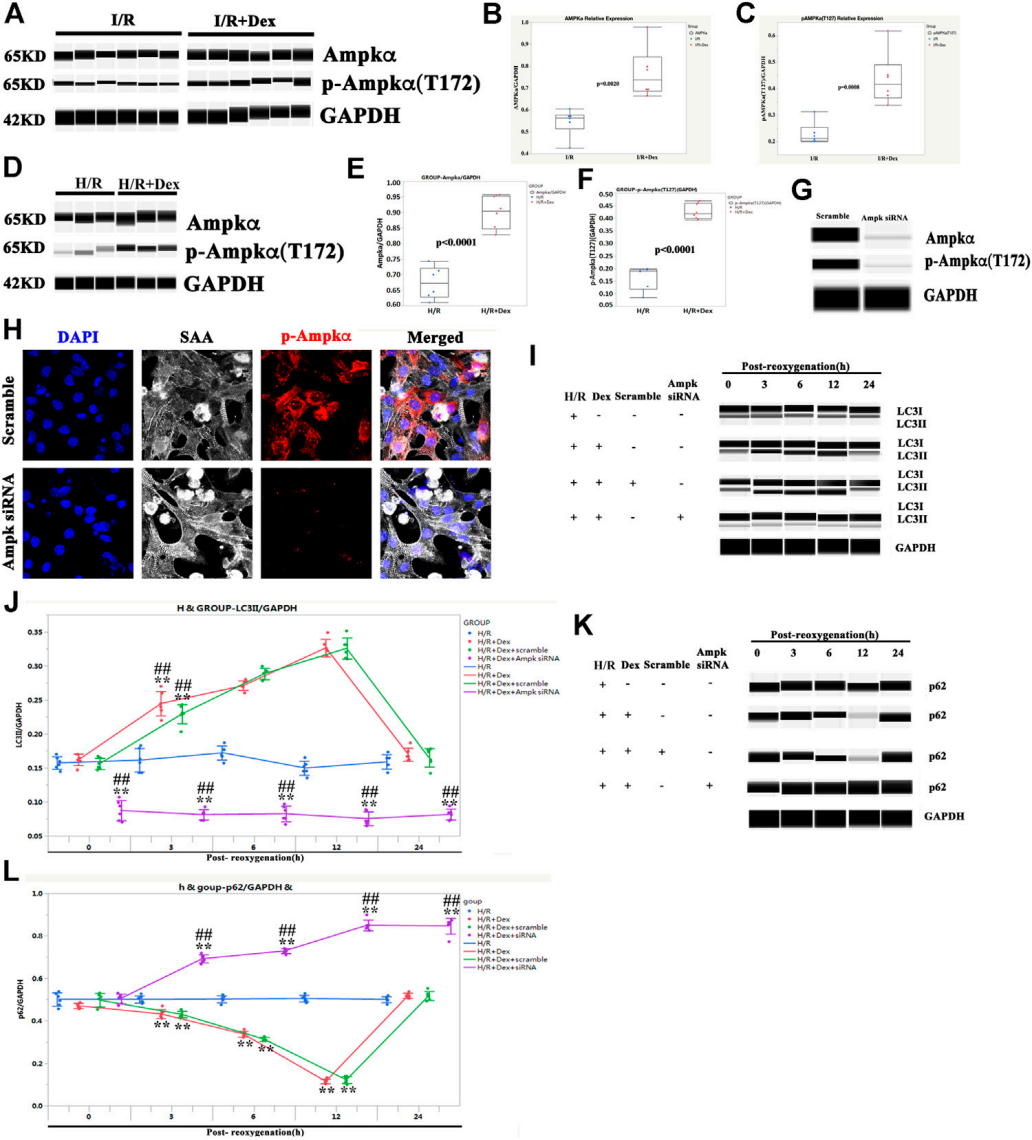
The autophagy induced by Dex was AMPK dependent. **(A)** Representative AMPK/p-AMPK Wes blot of a human ventricle pretreated with Dex. **(B)** Quantification of the AMPK relative expression of a human atria. N = 6 patients. **(C)** Quantification of the p-AMPK relative expression of a human ventricle. N = 6 patients. **(D)** Representative AMPK/p-AMPK Wes blot of iPSC-CMs pretreated with Dex. **(E)** Quantification of the AMPK relative expression of iPSC-CMs. N = 6 replicates. **(F)** Quantification of the p-AMPK relative expression of iPSC-CMs. N = 6 replicates. **(G)** The expression of AMPK and p-AMPK were reduced by AMPK siRNA, as indicated by the Wes blot. **(H)** The expression of p-AMPK was reduced by AMPK siRNA, as indicated by immunostaining. **(I)** AMPK siRNA blocked the increased expression of LC3II by Dex, as indicated by the Wes blot. **(J)** Quantification of the LC3II expression in Panel 6I. **(K)** AMPK siRNA blocked the reduced expression of p62 by Dex, as indicated by the Wes blot. **(L)** Quantification of the p62 expression in Panel 6K. ** *p* <0.01. vs. H/R; ## *p* <0.01, vs. H/R + Dex.

As it was previously shown that Dex increased the expression of AMPK in a mouse I/R model (Sun et al., 2017) and that AMPK was required for targeting mitochondria to lysosomes for autophagy degradation (Laker et al., 2017), it was sought to determine whether the autophagy induced by Dex in human atria, which causes I/R protection, was AMPK dependent. As shown in Figures 6A–C, the Dex pretreatment significantly increased the expression of AMPK and p-AMPK in human atria samples after the I/R process. Similarly, the expressions of AMPK and p-AMPK in iPSC-CMs were increased at the time of post-oxygenation (12 h) after the Dex pretreatment (Figures 6D–F).

To confirm the role of AMPK in Dex protection, AMPK was knocked down (Figures 6G,H), and the iPSC-CMs were treated with Dex again. The LC3II expression in the AMPK siRNA group was downregulated at the beginning of reoxygenation and lasted for 24 h after reoxygenation compared with the H/R group (Figures 6I,J). The effect of Dex on the LC3II expression was also blocked when AMPK was knocked down (Figures 6I,J). The expression of p62 showed a reverse trend compared with LC3II (Figures 6K,L). These results indicated that the autophagy induced by Dex was AMPK dependent.

The protection of Dex on H/R injury was α2-adrenergic receptor dependent

**FIGURE 7:**
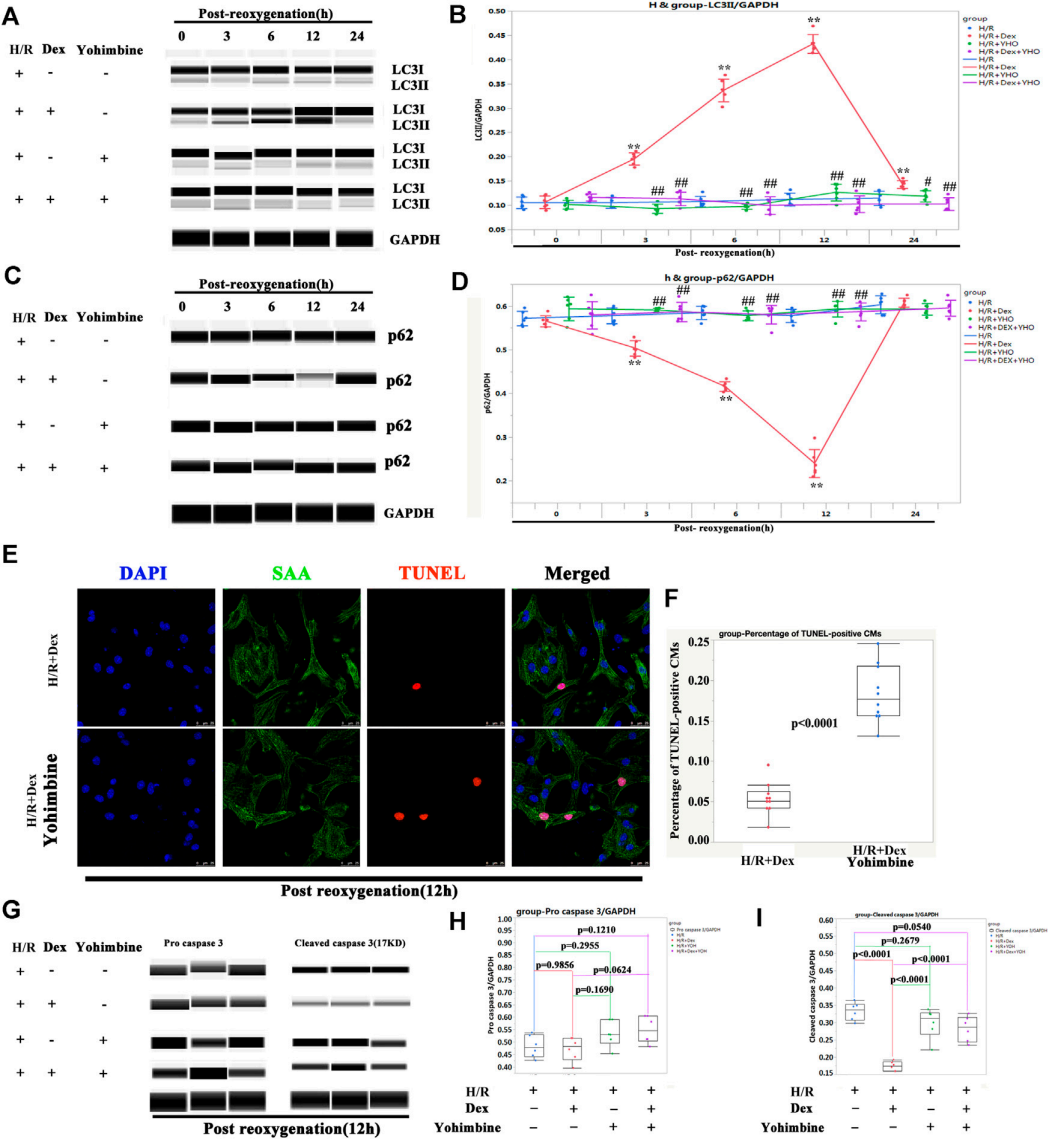
The protection of Dex was α2-adrenergic receptor dependent. **(A)** Representative LC3II Wes blot of iPSC-CMs pretreated with Dex and yohimbine (α2-adrenergic receptor antagonist). **(B)** Quantification of the LC3II relative expression. N = 3 independent experiments. **(C)** Representative p62 Wes blot of iPSC-CMs pretreated with Dex and yohimbine. **(D)** Quantification of the p62 relative expression. N = 3 independent experiments. **(E)** Representative TUNEL-positive CMs after treatment with Dex and yohimbine during the H/R process. **(F)** Quantification of the TUNEL-positive CMs. N = 10 fields. **(G)** Representative pro-cleaved caspase 3 Wes blot of iPSC-CMs pretreated with Dex and yohimbine. **(H)** Quantification of the pro-caspase 3 relative expression. N = 6 replicates. **(I)** Quantification of the cleaved caspase 3 relative expression. N = 6 replicates. ** *p* <0.01. vs. H/R; ## *p* <0.01, vs. H/R + Dex.

Since Dex is a highly selective α2-adrenergic receptor agonist, it was then investigated whether the protection of Dex on H/R injury was α2-adrenergic receptor dependent. The iPSC-CMs were pretreated with yohimbine (a α2-adrenergic receptor antagonist) 2 h before H/R. As shown in Figures 7A,B, Dex significantly increased the expression of LC3II after 3 h of reoxygenation and reached a peak at 12 h after reoxygenation. Yohimbine blocked the effect of Dex (Figures 7A,B). The expression of p62 showed a reverse trend compared with LC3II (Figures 7C,D). As a result, the TUNEL-positive CMs were significantly increased in the H/R + Dex + yohimbine group as compared to the H/R + Dex group (Figures 7E,F). The Wes results showed that the reduced expression of cleaved caspase 3 caused by Dex was reversed by the yohimbine (Figures 7G–I). These results demonstrated that the protection of Dex for H/R injury was α2-adrenergic receptor dependent.

## Discussion

The incidence of CHDs in China has reached 7 per 1,000, of which complicated CHD cases account for 30–40%. CHD is the leading cause of non-accidental death in children under 5 years of age (Zhao et al., 2020). Most CHD cases require surgical treatment. With the rapid development of medical technology, the efficacy of surgical treatment for CHD has significantly improved, but I/R injury during the surgical process still poses a severe threat to child health (Senst et al., 2020). Therefore, determining how to effectively control I/R injury after CPB in infants and young children has become an important medical problem. The current study first demonstrated that Dex was effective for protecting young human CMs from I/R injury *in vivo* and H/R injury *in vitro*. Thus, Dex and its mimics may provide hope for improvements in postsurgical CHD treatment.

The current rodent I/R models have only been applied to adult animals because the surgical process of neonatal myocardium infarction (MI) is quite different from the process in adults (Ponnusamy et al., 2019; Wang et al., 2019; Zlatanova et al., 2019). During the neonatal MI process, pups need to be put on an ice bed for the operation. It is impossible to place pups on an ice bed for 4–6 h and then reopen the coronary left anterior descending branch because the pups will die when placed on an ice bed for more than 30 min. Due to the significant differences between neonatal and adult cardiomyocytes, the results obtained from adult CMs cannot be applied directly to the neonatal heart (Sun et al., 2019; Yokota et al., 2020). In agreement with this, many drugs effective for adult human heart failure therapy are ineffective for infant heart failure therapy (Alabed et al., 2016; Rasool et al., 2016). In this study, surgically removed tissues were utilized to mimic the I/R process. The CPB phase mimicked the ischemia phase, and the oxygen-rich environment culture phase mimicked the reperfusion phase. This was the first time that a new and young human I/R model has been introduced, and this model can be used for the initial assessment of other drugs as well.

Autophagy has attracted much attention in recent years (Zhang et al., 2017; Li et al., 2020; Mookherjee et al., 2020). Li *et al.* showed that autophagy protected the heart from I/R injury *via* apoptosis associated protein recruitment (Li et al., 2020). In the current study, it was first demonstrated that the protection of Dex in human I/R was associated with autophagy, which was α2-adrenergic receptor/AMPK dependent. However, it should be noted that excessive autophagy is detrimental to CMs (Davidson et al., 2020; Fernández et al., 2020). How Dex regulates autophagy to protect CMs from I/R injury requires further investigation. Another limitation of the study is that the original human sample was under hypoxia (Table 1). Whether or not this precondition interfered with the study’s results remains unclear.

Although the current study showed that the α2-adrenergic receptor agonist, Dex, induced the expression of AMPK under the condition of I/R (Figures 6A–F) and the effect of Dex on the expression of AMPK was similar in a mouse I/R model (Sun et al., 2017), how the α2-adrenergic receptor stimulation regulated the AMPK activity requires more investigation. A possible connection is oxidative stress, which is involved in both Dex functions and AMPK activities (Riquelme et al., 2016; Ma et al., 2020 Jan). In addition, it was shown that Dex protects mice against I/R injury by activating the AMPK/PI3K/Akt/eNOS pathway (Sun et al., 2017). PI3K/Akt is a primary core and downstream point in the signaling pathway network (Sun et al., 2017). Many signaling pathways are connected by PI3K/Akt (Sun et al., 2017). PI3K/Akt may be the connection of AMPK and the α2-adrenergic receptor (Supplementary Figure S2).

There remains the question of why no other α2-adrenergic receptor agonists (e.g. norepinephrine) have been found to protect the heart from I/R injury. It is possible that α2‐adrenergic receptor-dependence is a necessary condition but not a sufficient condition. Previous publications showed that the anti-inflammatory and anti-oxidative effects of Dex were α2‐adrenergic-receptor-dependent (Gao et al., 2019 Dec). There are three a2-adrenergic receptor subtypes, all of which couple to multiple effectors *via* Gi/Go proteins. They perform various functions, including the mediation of decreases in adenylyl cyclase activity, activation of receptor-mediated K+channels, and inhibition of voltage-gated Ca2+channels. There are pairs of Gi/Go proteins, distributed differently across different tissues (Saunders and Limbird, 1999 Nov). Different combinations of receptor subtypes and G proteins may be responsible for the different effects of α2‐adrenergic agonist.

Another concern is how the *in vitro* H/R process imitates the *in vivo* I/R process. According to current publications, the H/R models used for *in vitro* study have varied (Riquelme et al., 2016; Wang et al., 2018; Chu et al., 2019; Fernández et al., 2020; Ma et al., 2020 Jan). They can be divided into two categories. One is physical hypoxia, which places the cells under a hypoxic condition (Wang et al., 2018; Chu et al., 2019; Ma et al., 2020 Jan). The other is chemical hypoxia, in which oxygen scavengers deplete oxygen (Li et al., 2018; Gao et al., 2020). The primary purpose of the H/R process is to produce ROS, and all of the above models produce ROS, although the degree may vary (Wang et al., 2018; Chu et al., 2019; Ma et al., 2020 Jan). In the H/R model, the degree of hypoxia is another factor that should be considered. In previous work, it was shown that the degree of hypoxia affected the responses of iPSC-CMs (Ye et al., 2020). Other publications have assessed the effect of Dex on the I/R set cardiomyocyte *in vitro* at 1% hypoxia (Chang et al., 2020), and in order to be consistent with other publications, the iPSC-CMs were established to be under 1% O_2_ hypoxia.

In summary, this was the first study to demonstrate that Dex is effective in human heart I/R injury protection. The study also provided a model for evaluating I/R injury in human samples. Finally, it was demonstrated that α2-adrenergic receptor/AMPK-dependent autophagy may be one of the mechanisms by which Dex protects young human heart tissues from I/R injury.

## Data Availability

The original contributions presented in the study are included in the article/Supplementary Material, further inquiries can be directed to the corresponding authors.
