# Supplementary figures and images for: Dexmedetomidine Protects Human Cardiomyocytes Against Ischemia-Reperfusion Injury Through α2-Adrenergic Receptor/AMPK-Dependent Autophagy
Source: Front Pharmacol. 2021 May 21;12:615424. doi: 10.3389/fphar.2021.615424 (PMC8176440; doi:10.3389/fphar.2021.615424)

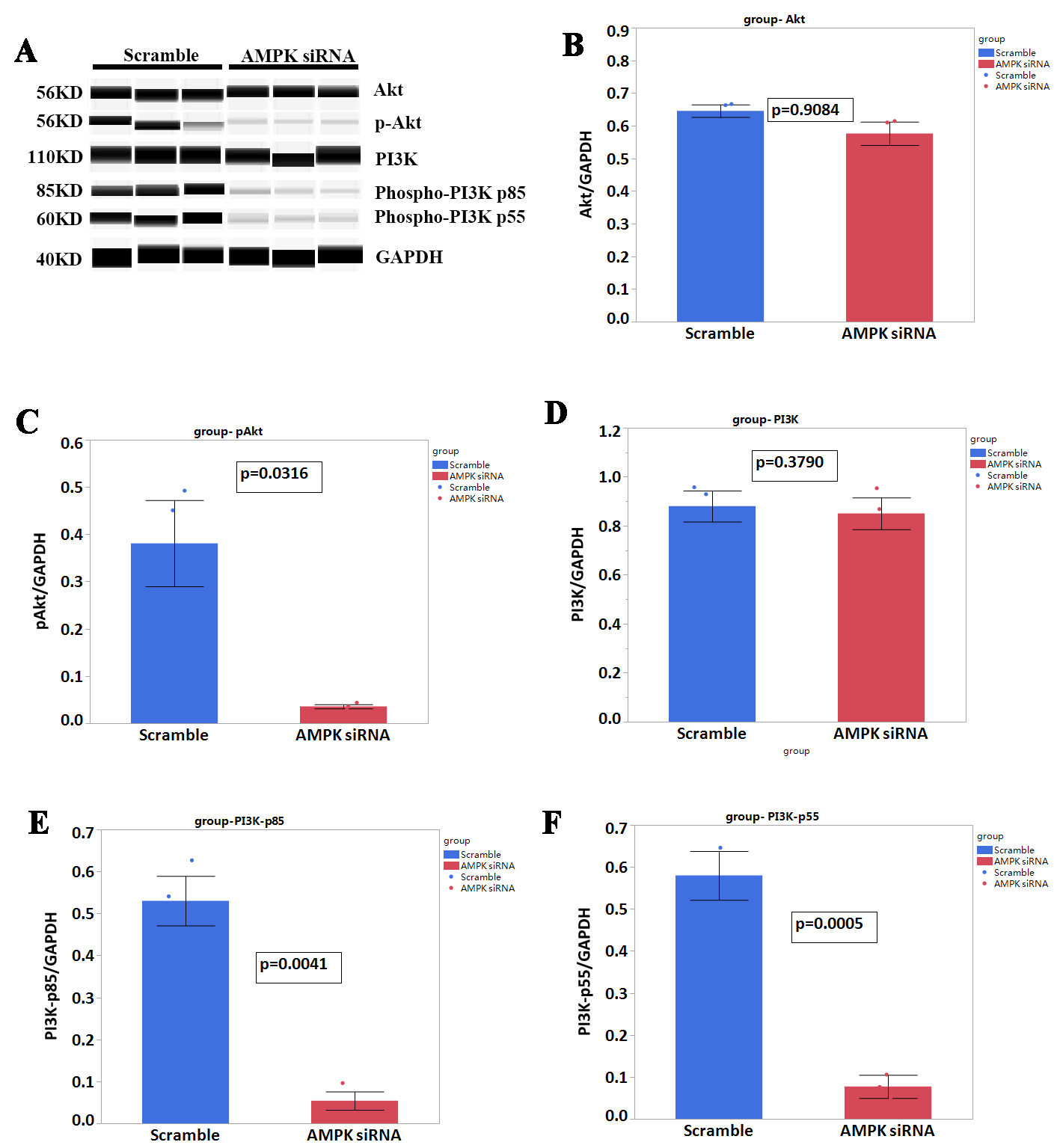

Supplement: Supplementary file 1 [file Image2.JPEG]
