# Supplementary material for: Dexmedetomidine Protects Human Cardiomyocytes Against Ischemia-Reperfusion Injury Through α2-Adrenergic Receptor/AMPK-Dependent Autophagy
Source: Front Pharmacol. 2021 May 21;12:615424. doi: 10.3389/fphar.2021.615424 (PMC8176440; doi:10.3389/fphar.2021.615424)
Supplement: Supplementary file 3 [file DataSheet1.PDF]

Fig.1D

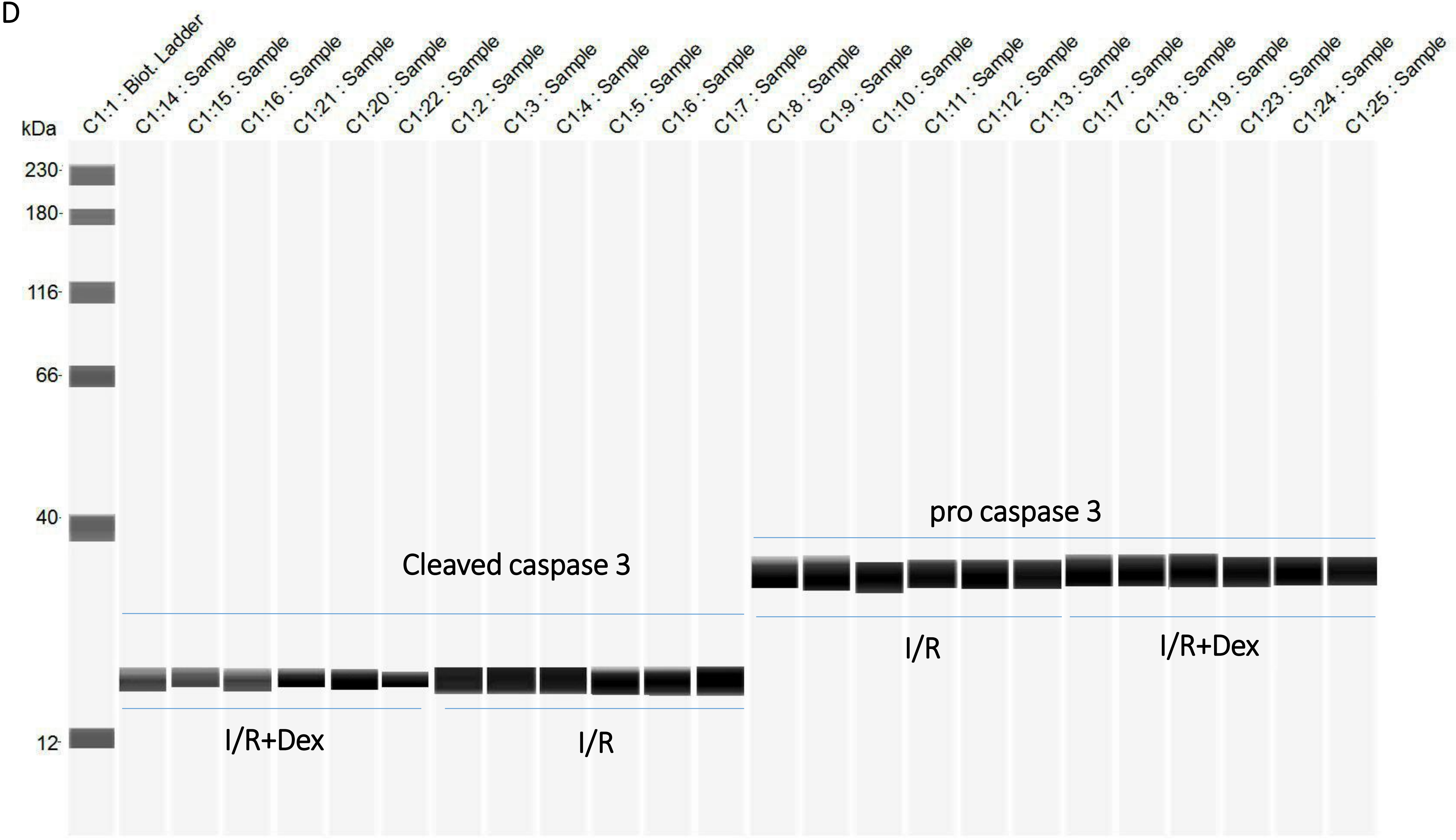
kDa
C1:1 : Biot. Ladder
C1:14 : Sample
C1:15 : Sample
C1:16 : Sample
C1:21 : Sample
C1:20 : Sample
C1:22 : Sample
C1:2 : Sample
C1:3 : Sample
C1:4 : Sample
C1:5 : Sample
C1:6 : Sample
C1:7 : Sample
C1:8 : Sample
C1:9 : Sample
C1:10 : Sample
C1:11 : Sample
C1:12 : Sample
C1:13 : Sample
C1:17 : Sample
C1:18 : Sample
C1:19 : Sample
C1:23 : Sample
C1:24 : Sample
C1:25 : Sample
230
180
116
66
40
12
pro caspase 3
Cleaved caspase 3
I/R
I/R+Dex
I/R+Dex
I/R

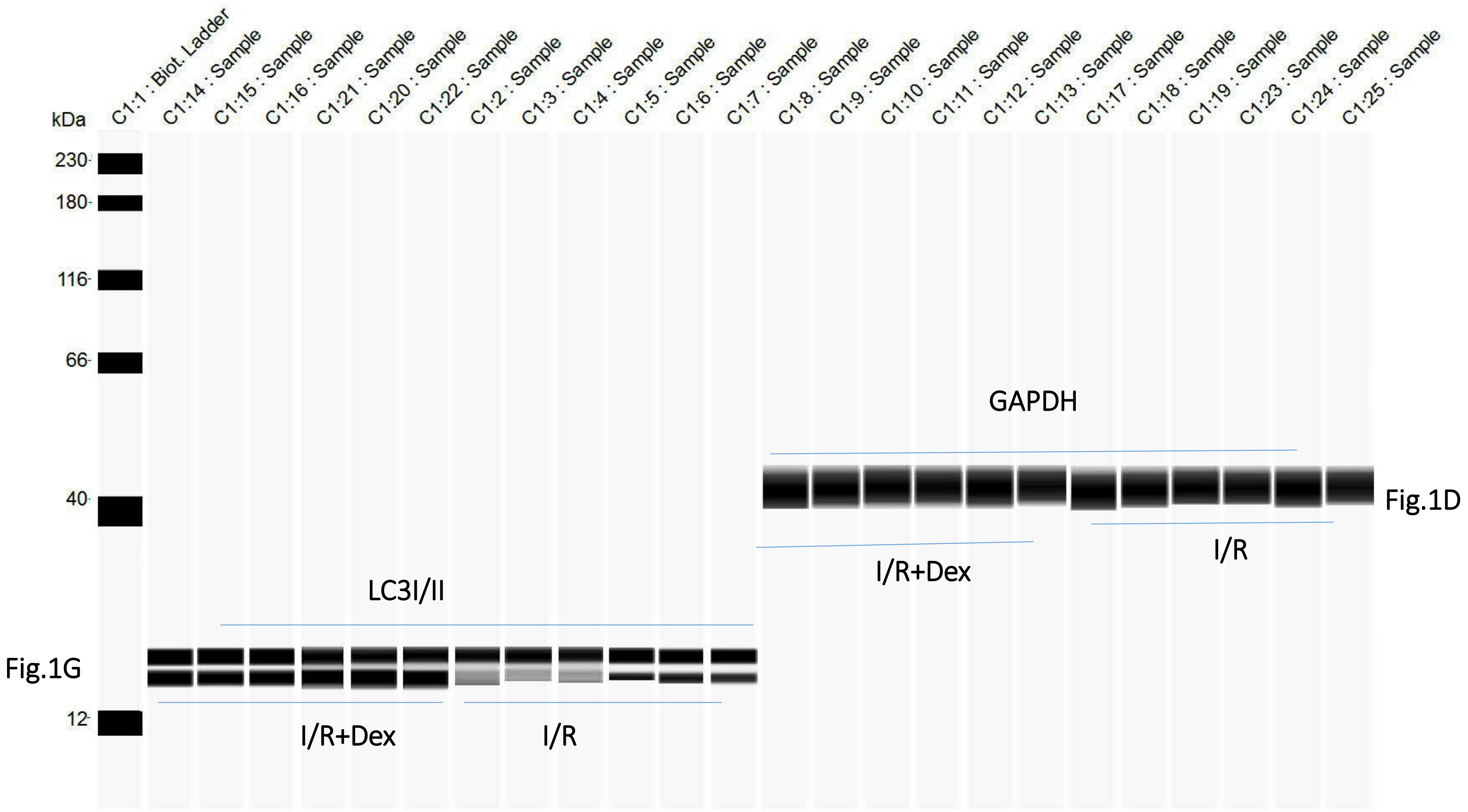

kDa
C1:1 : Biot. Ladder
C1:14 : Sample
C1:15 : Sample
C1:16 : Sample
C1:21 : Sample
C1:20 : Sample
C1:22 : Sample
C1:2 : Sample
C1:3 : Sample
C1:4 : Sample
C1:5 : Sample
C1:6 : Sample
C1:7 : Sample
C1:8 : Sample
C1:9 : Sample
C1:10 : Sample
C1:11 : Sample
C1:12 : Sample
C1:13 : Sample
C1:17 : Sample
C1:18 : Sample
C1:19 : Sample
C1:23 : Sample
C1:24 : Sample
C1:25 : Sample
230
180
116
66
40
12
GAPDH
Fig.1D
I/R+Dex
I/R
LC3I/II
Fig.1G
I/R+Dex
I/R

Fig.1G

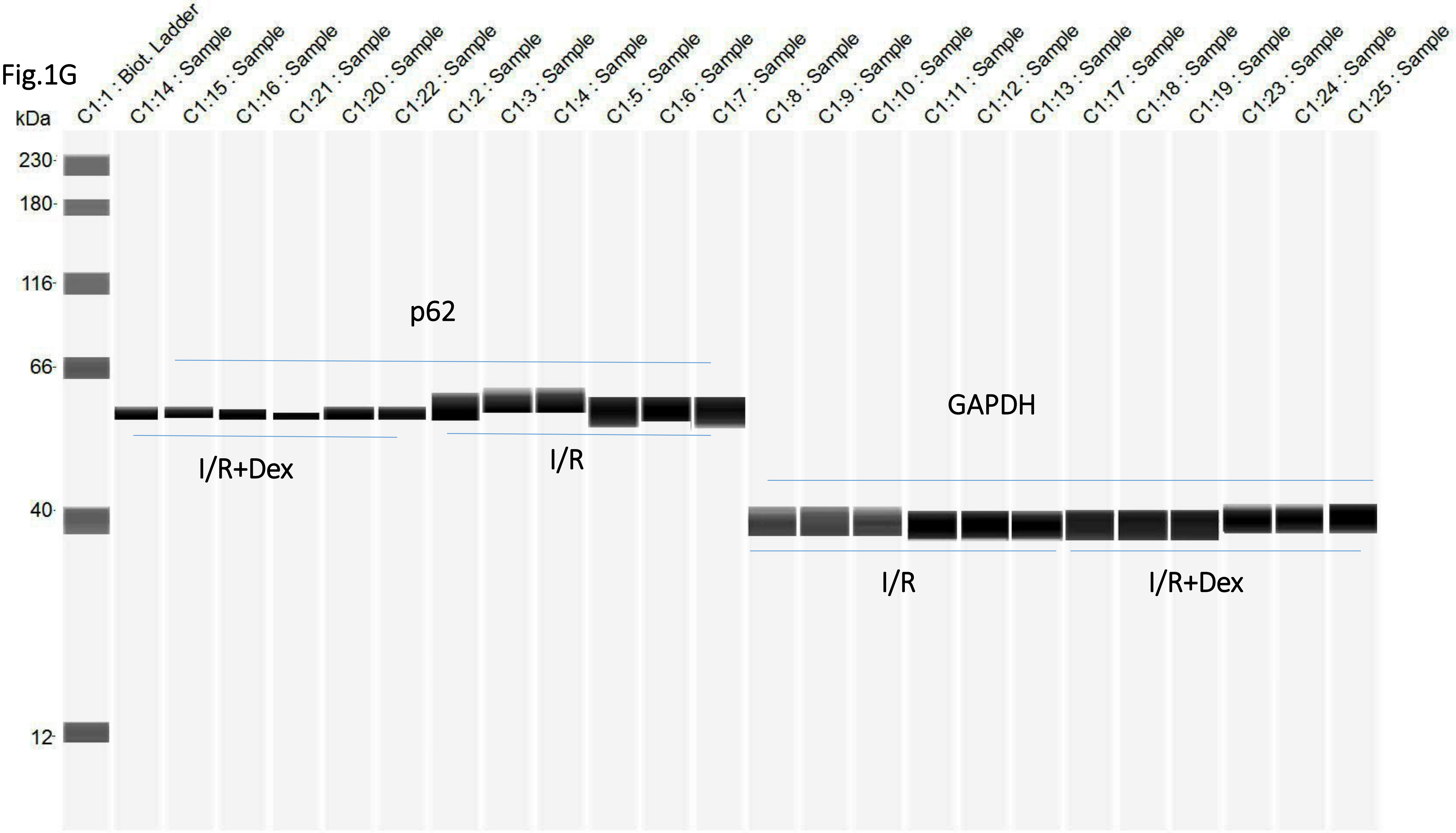

kDa
C1:1 : Biot. Ladder
C1:14 : Sample
C1:15 : Sample
C1:16 : Sample
C1:21 : Sample
C1:20 : Sample
C1:22 : Sample
C1:2 : Sample
C1:3 : Sample
C1:4 : Sample
C1:5 : Sample
C1:6 : Sample
C1:7 : Sample
C1:8 : Sample
C1:9 : Sample
C1:10 : Sample
C1:11 : Sample
C1:12 : Sample
C1:13 : Sample
C1:17 : Sample
C1:18 : Sample
C1:19 : Sample
C1:23 : Sample
C1:24 : Sample
C1:25 : Sample
230
180
116
66
40
12
p62
I/R+Dex
I/R
GAPDH
I/R
I/R+Dex

Fig.2D

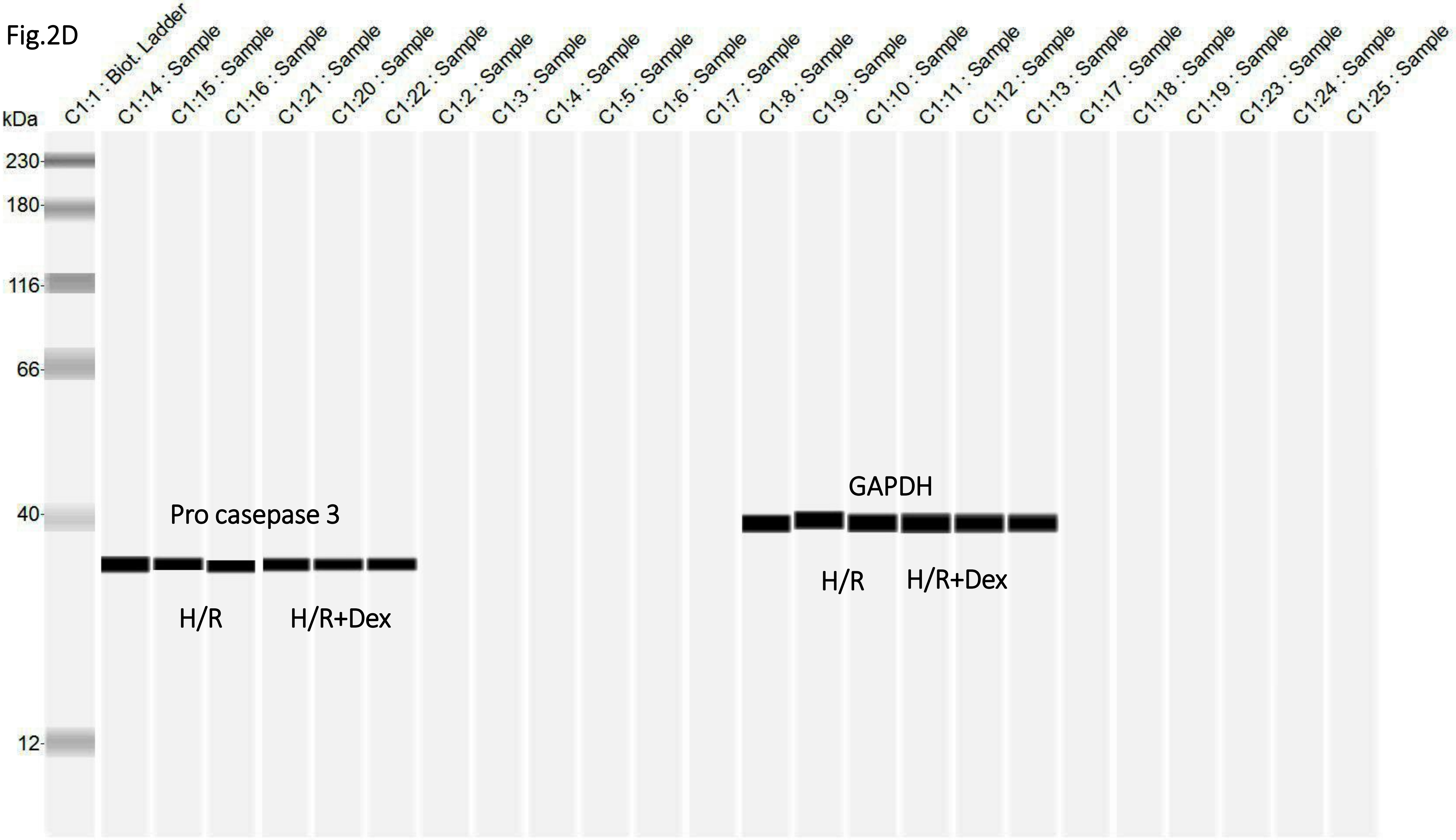

C1:1 : Biot. Ladder
C1:14 : Sample
C1:15 : Sample
C1:16 : Sample
C1:21 : Sample
C1:20 : Sample
C1:22 : Sample
C1:2 : Sample
C1:3 : Sample
C1:4 : Sample
C1:5 : Sample
C1:6 : Sample
C1:7 : Sample
C1:8 : Sample
C1:9 : Sample
C1:10 : Sample
C1:11 : Sample
C1:12 : Sample
C1:13 : Sample
C1:17 : Sample
C1:18 : Sample
C1:19 : Sample
C1:23 : Sample
C1:24 : Sample
C1:25 : Sample
kDa
230
180
116
66
40
12
Pro casepase 3
H/R
H/R+Dex
GAPDH
H/R
H/R+Dex

Fig.2G

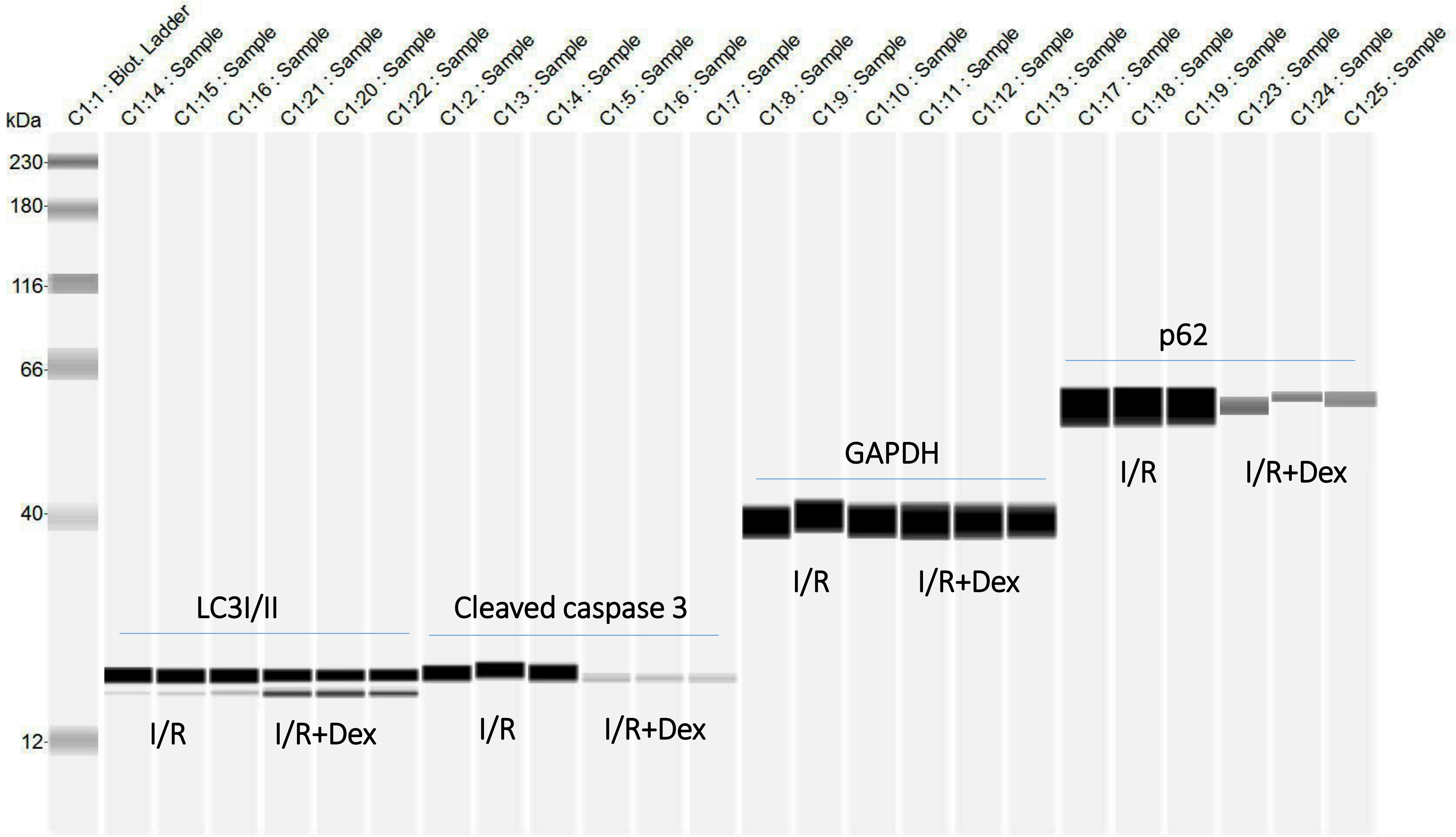
kDa
C1:1 : Biot. Ladder
C1:14 : Sample
C1:15 : Sample
C1:16 : Sample
C1:21 : Sample
C1:20 : Sample
C1:22 : Sample
C1:2 : Sample
C1:3 : Sample
C1:4 : Sample
C1:5 : Sample
C1:6 : Sample
C1:7 : Sample
C1:8 : Sample
C1:9 : Sample
C1:10 : Sample
C1:11 : Sample
C1:12 : Sample
C1:13 : Sample
C1:17 : Sample
C1:18 : Sample
C1:19 : Sample
C1:23 : Sample
C1:24 : Sample
C1:25 : Sample
230
180
116
66
40
12
p62
I/R
I/R+Dex
GAPDH
I/R
I/R+Dex
LC3I/II
Cleaved caspase 3
I/R
I/R+Dex
I/R
I/R+Dex

Fig.3A

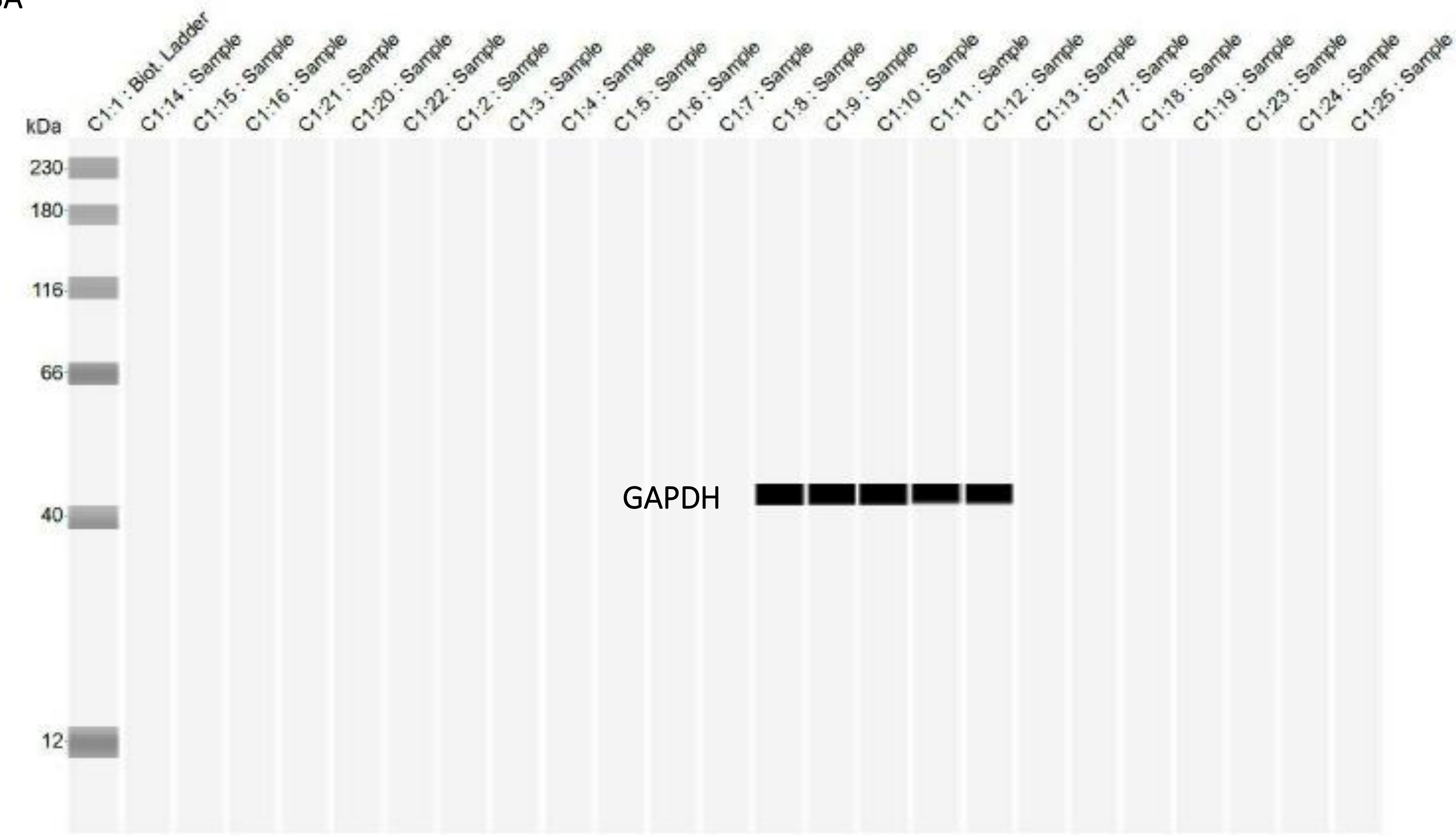
kDa
230
180
116
66
40
12
C1:1 : Biot. Ladder
C1:14 : Sample
C1:15 : Sample
C1:16 : Sample
C1:21 : Sample
C1:20 : Sample
C1:22 : Sample
C1:2 : Sample
C1:3 : Sample
C1:4 : Sample
C1:5 : Sample
C1:6 : Sample
C1:7 : Sample
C1:8 : Sample
C1:9 : Sample
C1:10 : Sample
C1:11 : Sample
C1:12 : Sample
C1:13 : Sample
C1:17 : Sample
C1:18 : Sample
C1:19 : Sample
C1:23 : Sample
C1:24 : Sample
C1:25 : Sample
GAPDH

Fig.3A

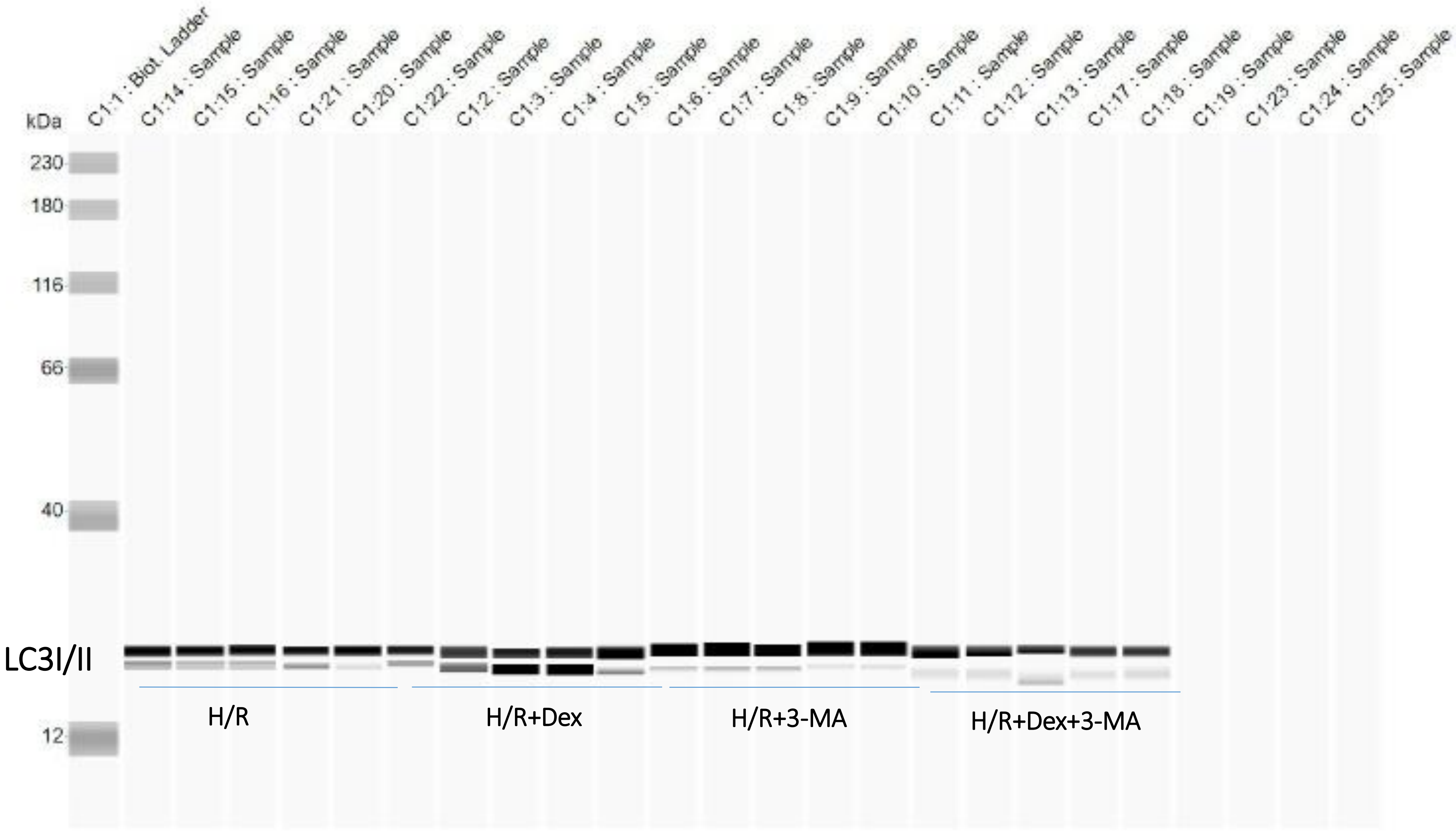

kDa
C1:1 : Biot. Ladder
C1:14 : Sample
C1:15 : Sample
C1:16 : Sample
C1:21 : Sample
C1:20 : Sample
C1:22 : Sample
C1:2 : Sample
C1:3 : Sample
C1:4 : Sample
C1:5 : Sample
C1:6 : Sample
C1:7 : Sample
C1:8 : Sample
C1:9 : Sample
C1:10 : Sample
C1:11 : Sample
C1:12 : Sample
C1:13 : Sample
C1:17 : Sample
C1:18 : Sample
C1:19 : Sample
C1:23 : Sample
C1:24 : Sample
C1:25 : Sample
230
180
116
66
40
12
LC3I/II
H/R
H/R+Dex
H/R+3-MA
H/R+Dex+3-MA

Fig.3C

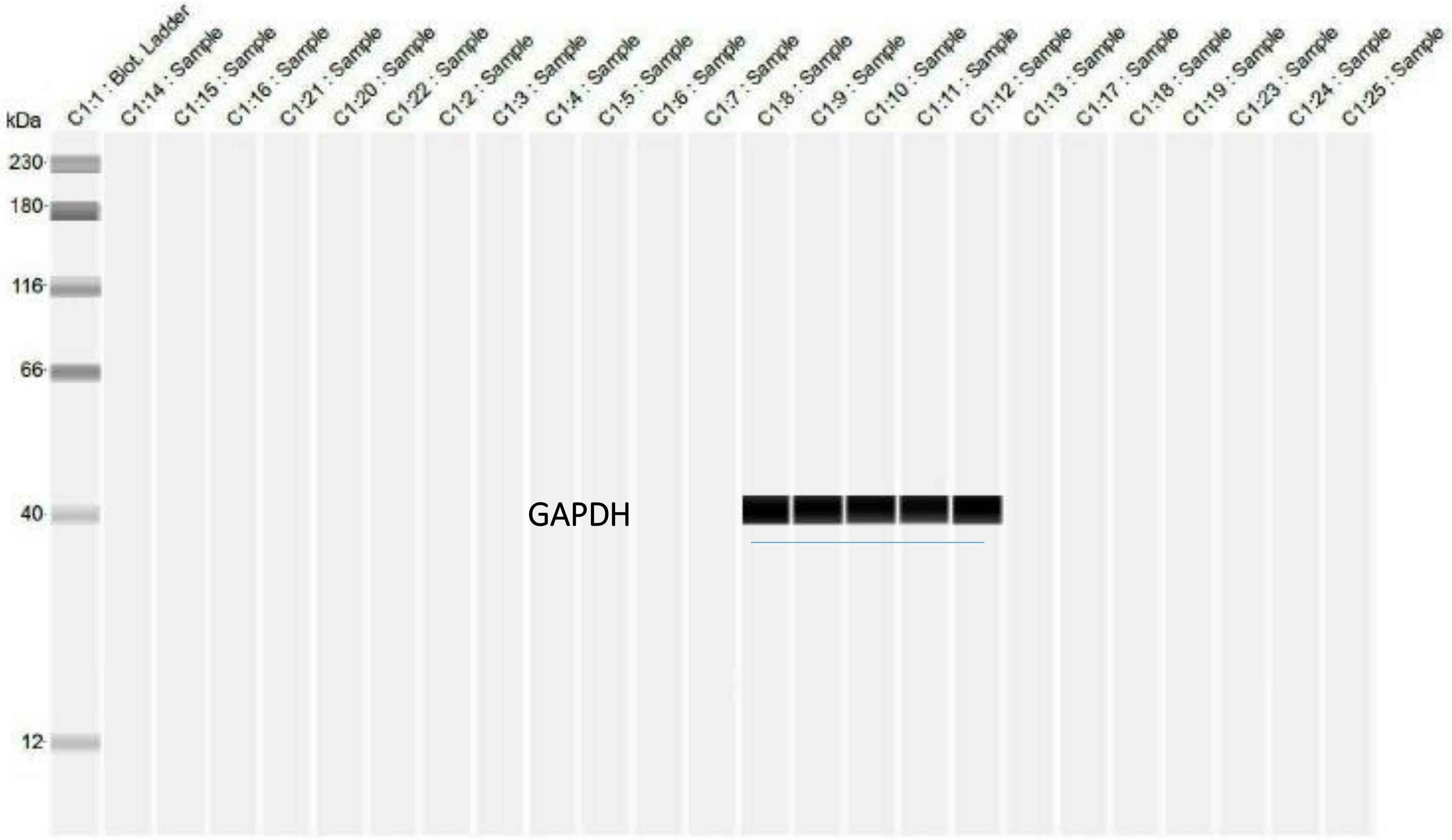

kDa
230
180
116
66
40
12
C1:1 : Biot. Ladder
C1:14 : Sample
C1:15 : Sample
C1:16 : Sample
C1:21 : Sample
C1:20 : Sample
C1:22 : Sample
C1:2 : Sample
C1:3 : Sample
C1:4 : Sample
C1:5 : Sample
C1:6 : Sample
C1:7 : Sample
C1:8 : Sample
C1:9 : Sample
C1:10 : Sample
C1:11 : Sample
C1:12 : Sample
C1:13 : Sample
C1:17 : Sample
C1:18 : Sample
C1:19 : Sample
C1:23 : Sample
C1:24 : Sample
C1:25 : Sample
GAPDH

Fig.3C

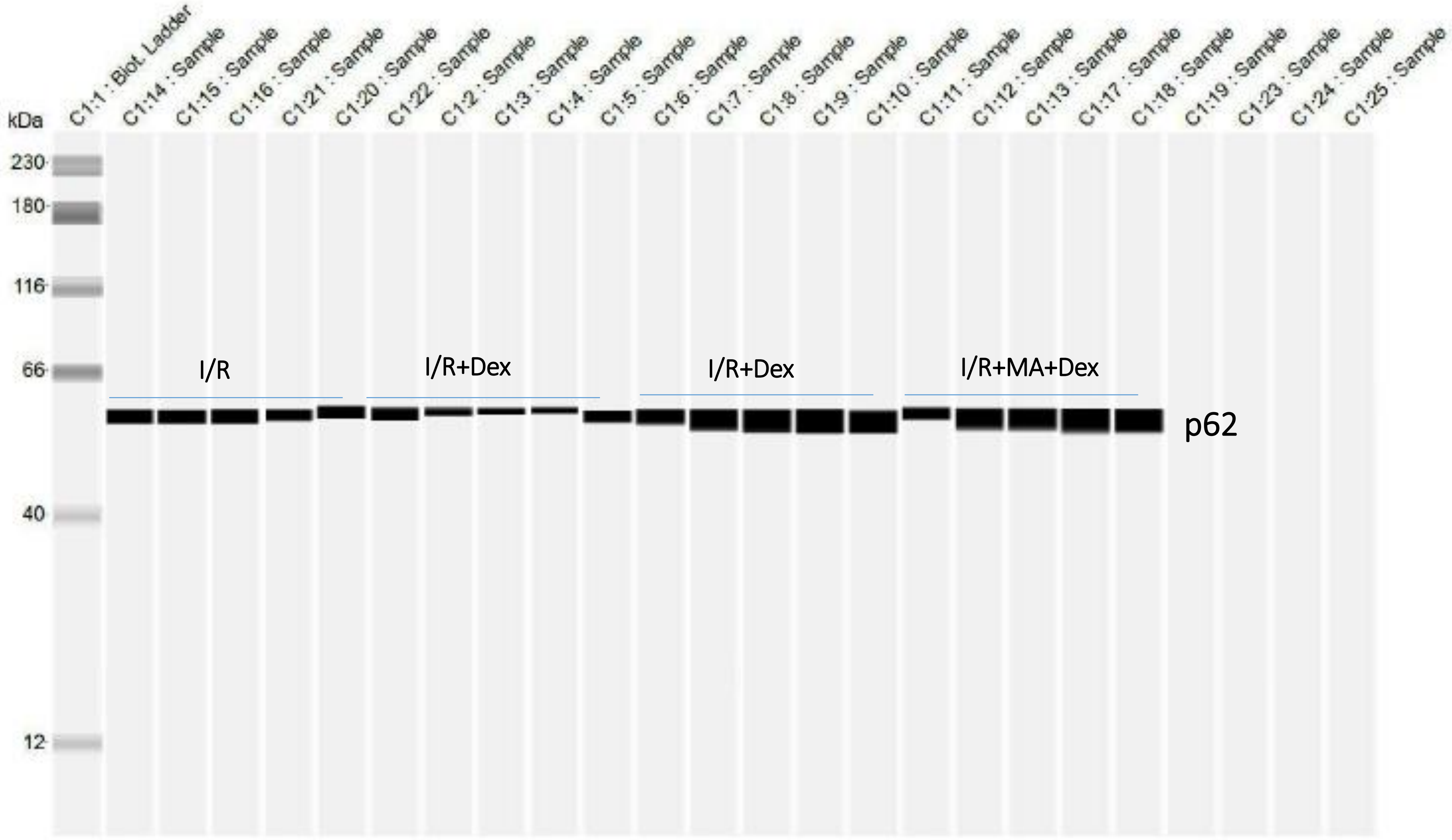

C1:1 : Biot. Ladder
C1:14 : Sample
C1:15 : Sample
C1:16 : Sample
C1:21 : Sample
C1:20 : Sample
C1:22 : Sample
C1:2 : Sample
C1:3 : Sample
C1:4 : Sample
C1:5 : Sample
C1:6 : Sample
C1:7 : Sample
C1:8 : Sample
C1:9 : Sample
C1:10 : Sample
C1:11 : Sample
C1:12 : Sample
C1:13 : Sample
C1:17 : Sample
C1:18 : Sample
C1:19 : Sample
C1:23 : Sample
C1:24 : Sample
C1:25 : Sample
kDa
230
180
116
66
40
12
I/R
I/R+Dex
I/R+Dex
I/R+MA+Dex
p62

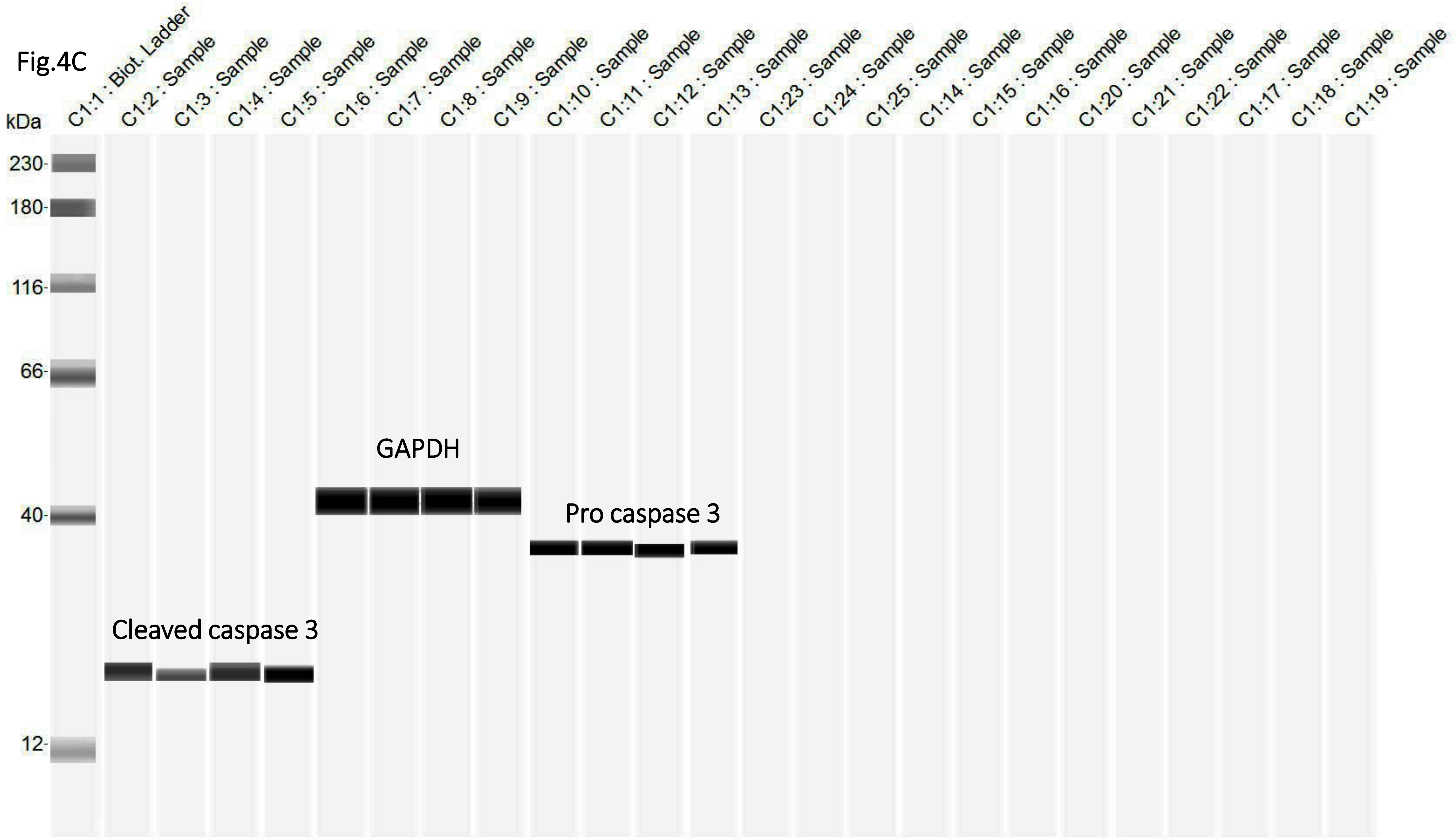
Fig.4C
C1:1 : Biot. Ladder
C1:2 : Sample
C1:3 : Sample
C1:4 : Sample
C1:5 : Sample
C1:6 : Sample
C1:7 : Sample
C1:8 : Sample
C1:9 : Sample
C1:10 : Sample
C1:11 : Sample
C1:12 : Sample
C1:13 : Sample
C1:23 : Sample
C1:24 : Sample
C1:25 : Sample
C1:14 : Sample
C1:15 : Sample
C1:16 : Sample
C1:20 : Sample
C1:21 : Sample
C1:22 : Sample
C1:17 : Sample
C1:18 : Sample
C1:19 : Sample
kDa
230-
180-
116-
66-
40-
12-
GAPDH
Pro caspase 3
Cleaved caspase 3

Fig.5A

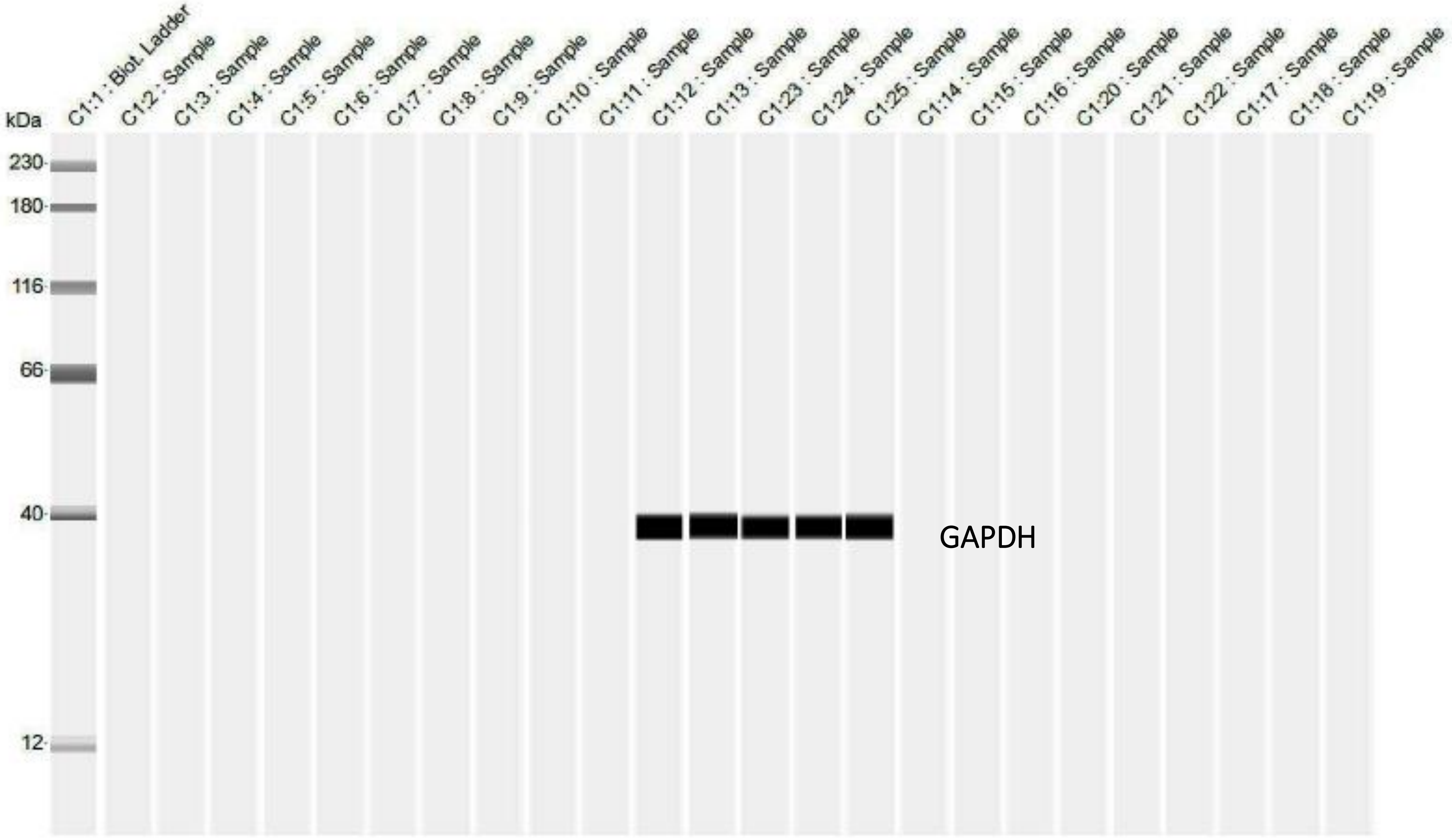
kDa
C1:1 : Biot. Ladder
C1:2 : Sample
C1:3 : Sample
C1:4 : Sample
C1:5 : Sample
C1:6 : Sample
C1:7 : Sample
C1:8 : Sample
C1:9 : Sample
C1:10 : Sample
C1:11 : Sample
C1:12 : Sample
C1:13 : Sample
C1:23 : Sample
C1:24 : Sample
C1:25 : Sample
C1:14 : Sample
C1:15 : Sample
C1:16 : Sample
C1:20 : Sample
C1:21 : Sample
C1:22 : Sample
C1:17 : Sample
C1:18 : Sample
C1:19 : Sample
230
180
116
66
40
12
GAPDH

Fig.5A

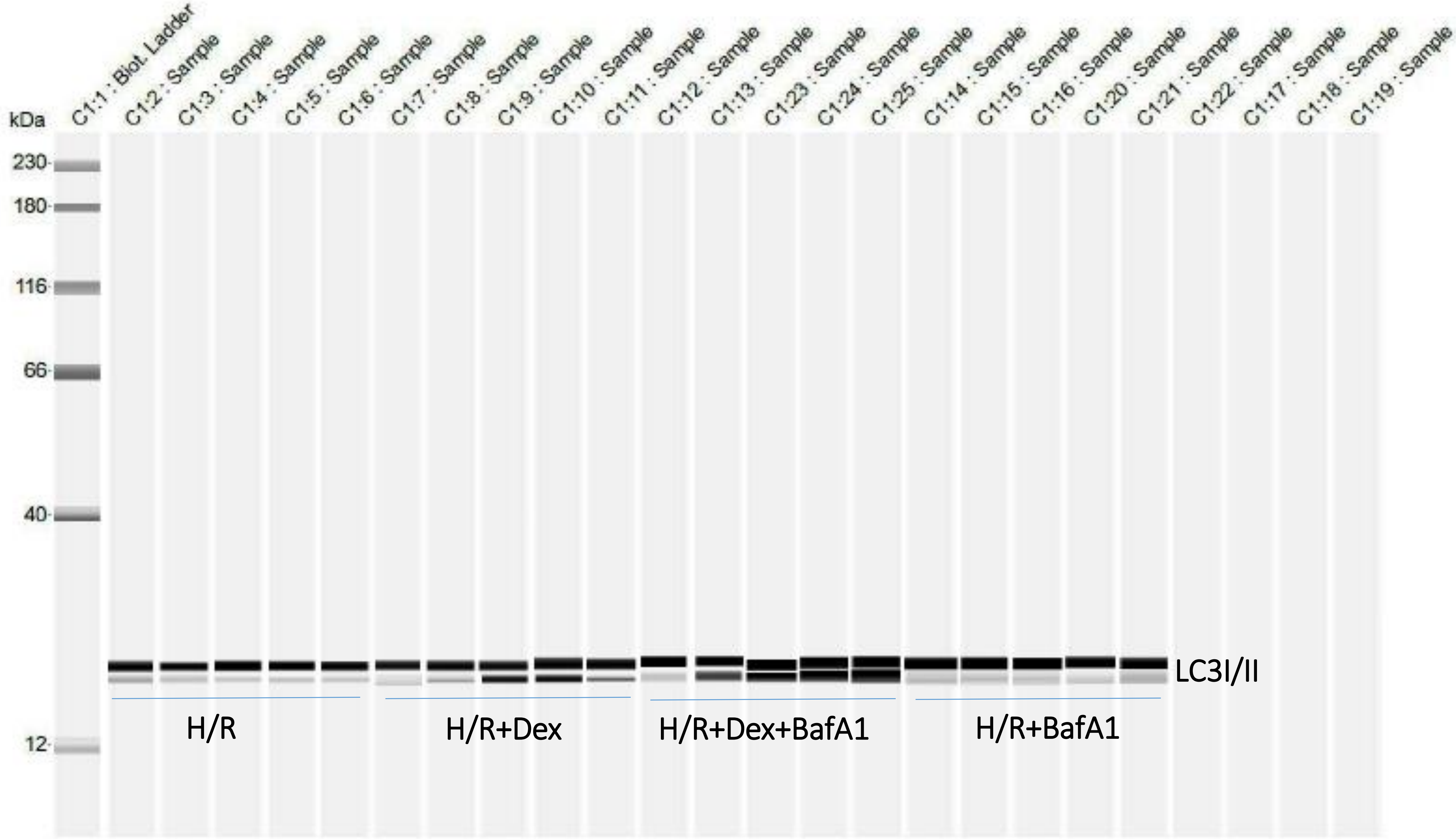

kDa
C1:1 : Biot. Ladder
C1:2 : Sample
C1:3 : Sample
C1:4 : Sample
C1:5 : Sample
C1:6 : Sample
C1:7 : Sample
C1:8 : Sample
C1:9 : Sample
C1:10 : Sample
C1:11 : Sample
C1:12 : Sample
C1:13 : Sample
C1:23 : Sample
C1:24 : Sample
C1:25 : Sample
C1:14 : Sample
C1:15 : Sample
C1:16 : Sample
C1:20 : Sample
C1:21 : Sample
C1:22 : Sample
C1:17 : Sample
C1:18 : Sample
C1:19 : Sample
230
180
116
66
40
12
LC3I/II
H/R
H/R+Dex
H/R+Dex+BafA1
H/R+BafA1

Fig.5C

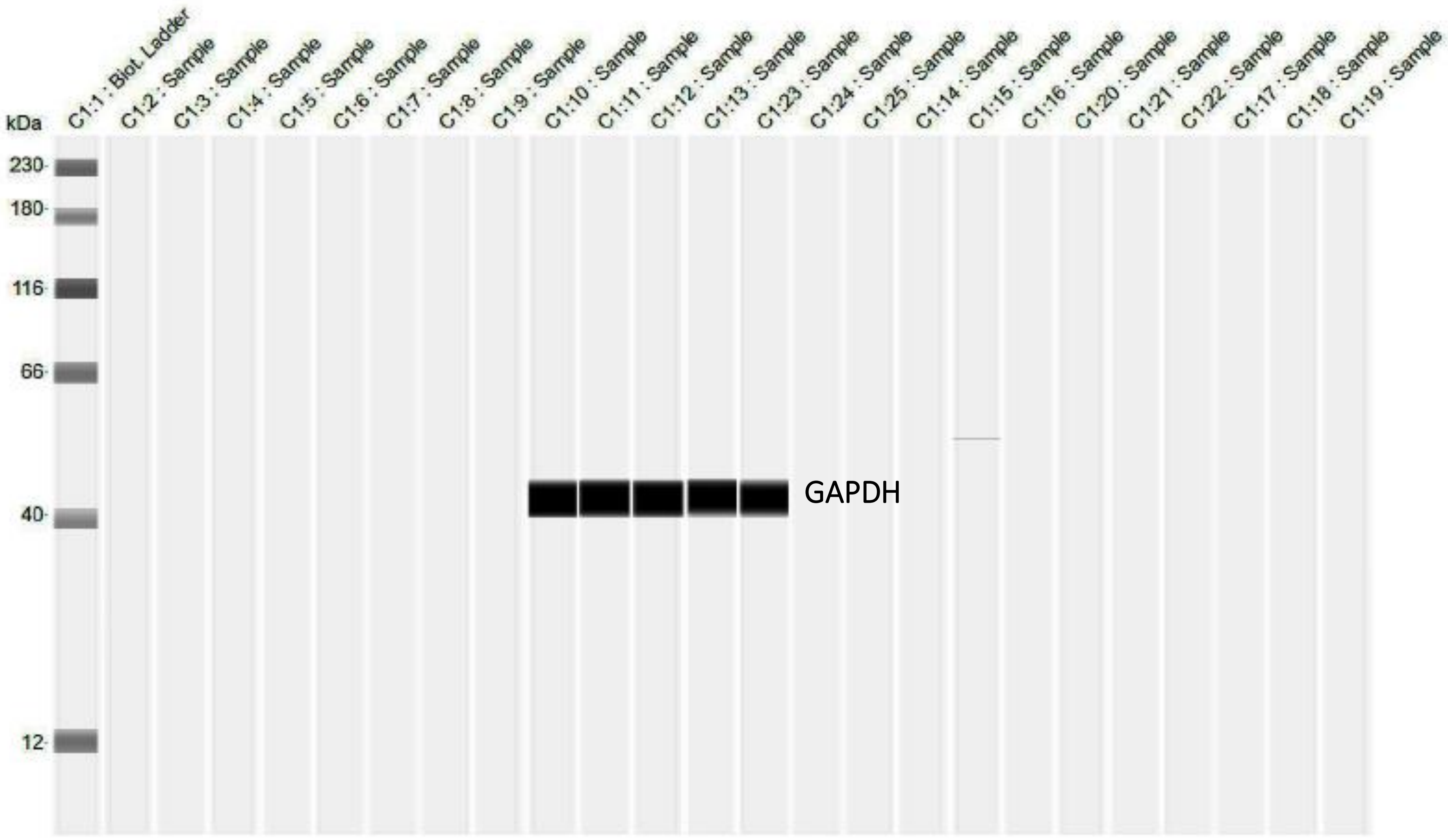
kDa
C1:1 : Biot. Ladder
C1:2 : Sample
C1:3 : Sample
C1:4 : Sample
C1:5 : Sample
C1:6 : Sample
C1:7 : Sample
C1:8 : Sample
C1:9 : Sample
C1:10 : Sample
C1:11 : Sample
C1:12 : Sample
C1:13 : Sample
C1:23 : Sample
C1:24 : Sample
C1:25 : Sample
C1:14 : Sample
C1:15 : Sample
C1:16 : Sample
C1:20 : Sample
C1:21 : Sample
C1:22 : Sample
C1:17 : Sample
C1:18 : Sample
C1:19 : Sample
230
180
116
66
40
12
GAPDH

Fig.5C

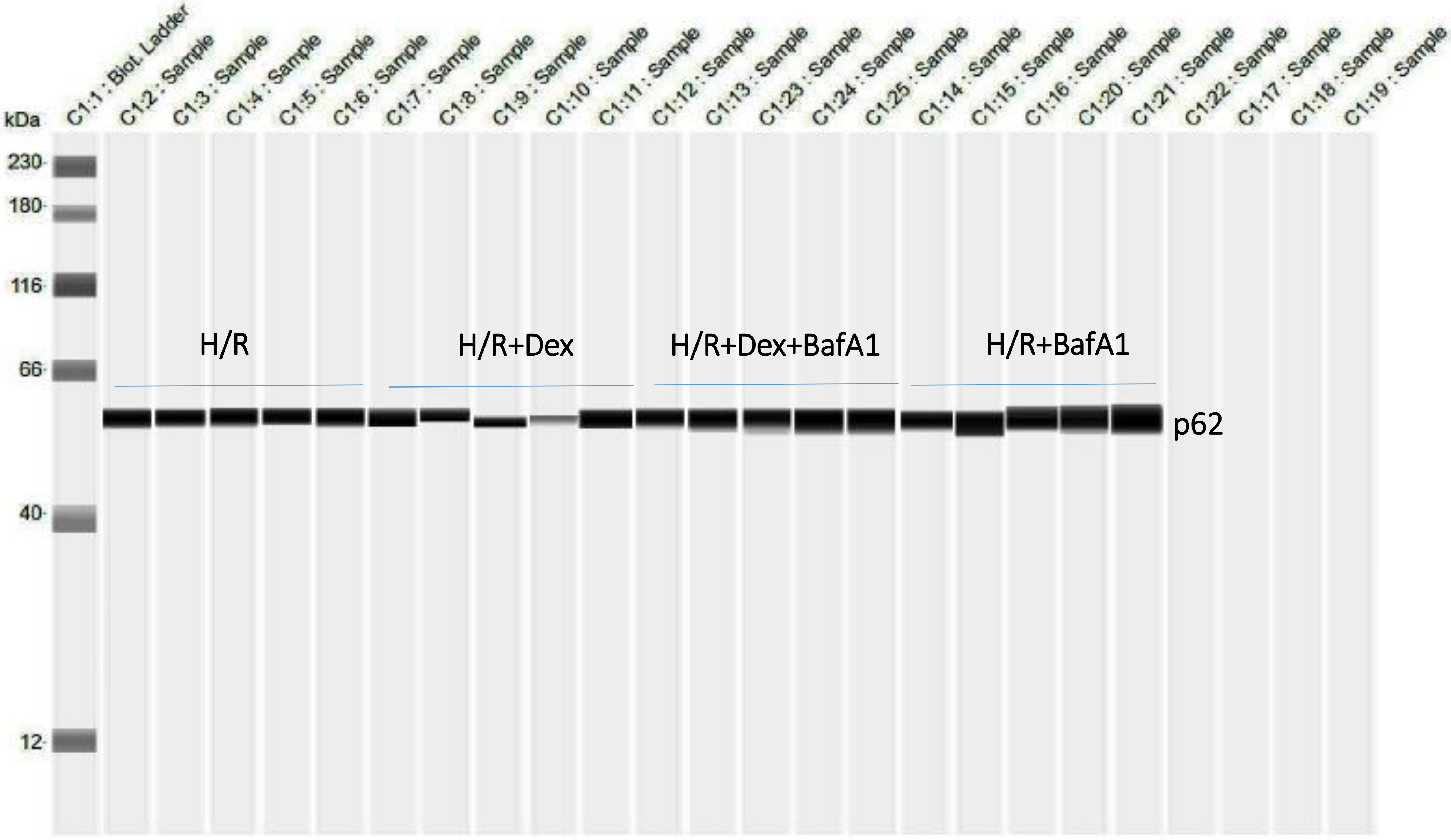

C1:1 : Biot. Ladder
C1:2 : Sample
C1:3 : Sample
C1:4 : Sample
C1:5 : Sample
C1:6 : Sample
C1:7 : Sample
C1:8 : Sample
C1:9 : Sample
C1:10 : Sample
C1:11 : Sample
C1:12 : Sample
C1:13 : Sample
C1:23 : Sample
C1:24 : Sample
C1:25 : Sample
C1:14 : Sample
C1:15 : Sample
C1:16 : Sample
C1:20 : Sample
C1:21 : Sample
C1:22 : Sample
C1:17 : Sample
C1:18 : Sample
C1:19 : Sample
kDa
230
180
116
66
40
12
H/R
H/R+Dex
H/R+Dex+BafA1
H/R+BafA1
p62

Fig.6A

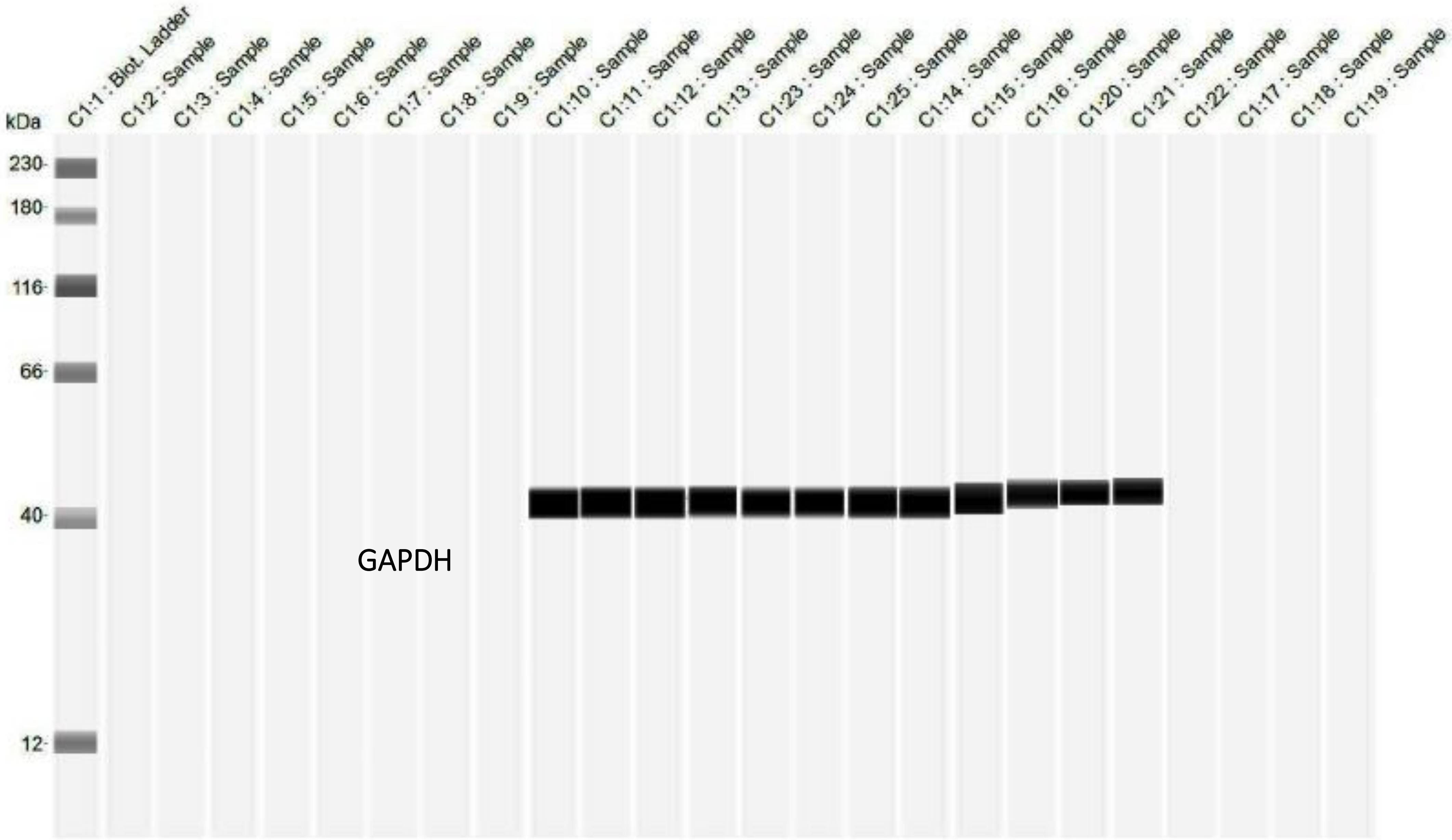
kDa
C1:1 : Biot. Ladder
C1:2 : Sample
C1:3 : Sample
C1:4 : Sample
C1:5 : Sample
C1:6 : Sample
C1:7 : Sample
C1:8 : Sample
C1:9 : Sample
C1:10 : Sample
C1:11 : Sample
C1:12 : Sample
C1:13 : Sample
C1:23 : Sample
C1:24 : Sample
C1:25 : Sample
C1:14 : Sample
C1:15 : Sample
C1:16 : Sample
C1:20 : Sample
C1:21 : Sample
C1:22 : Sample
C1:17 : Sample
C1:18 : Sample
C1:19 : Sample
230
180
116
66
40
12
GAPDH

Fig.6A

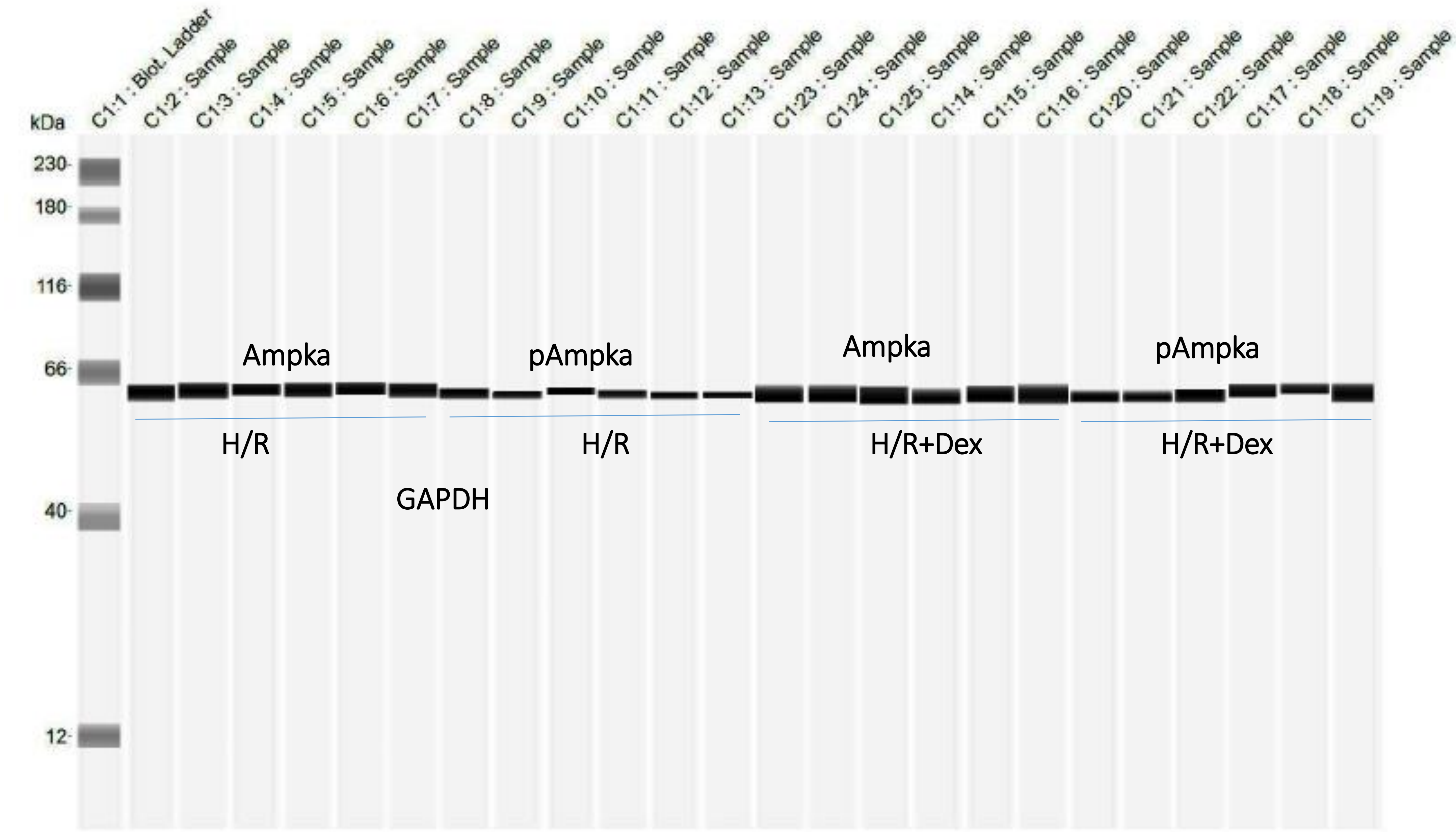
C1:1 : Biot. Ladder
C1:2 : Sample
C1:3 : Sample
C1:4 : Sample
C1:5 : Sample
C1:6 : Sample
C1:7 : Sample
C1:8 : Sample
C1:9 : Sample
C1:10 : Sample
C1:11 : Sample
C1:12 : Sample
C1:13 : Sample
C1:23 : Sample
C1:24 : Sample
C1:25 : Sample
C1:14 : Sample
C1:15 : Sample
C1:16 : Sample
C1:20 : Sample
C1:21 : Sample
C1:22 : Sample
C1:17 : Sample
C1:18 : Sample
C1:19 : Sample
kDa
230
180
116
66
40
12
Ampka
pAmpka
Ampka
pAmpka
H/R
H/R
H/R+Dex
H/R+Dex
GAPDH

Fig.6D

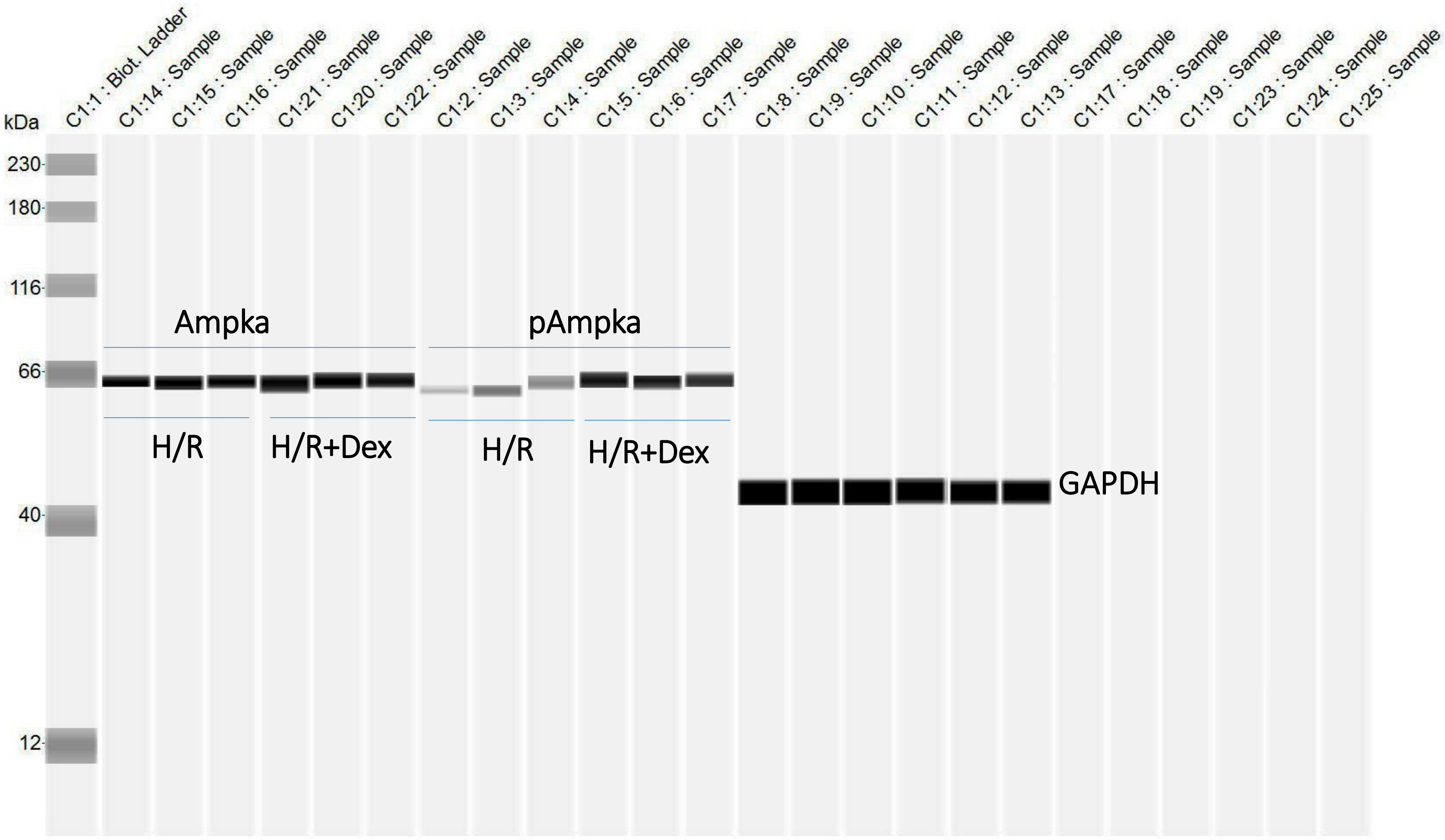

kDa
C1:1 : Biot. Ladder
C1:14 : Sample
C1:15 : Sample
C1:16 : Sample
C1:21 : Sample
C1:20 : Sample
C1:22 : Sample
C1:2 : Sample
C1:3 : Sample
C1:4 : Sample
C1:5 : Sample
C1:6 : Sample
C1:7 : Sample
C1:8 : Sample
C1:9 : Sample
C1:10 : Sample
C1:11 : Sample
C1:12 : Sample
C1:13 : Sample
C1:17 : Sample
C1:18 : Sample
C1:19 : Sample
C1:23 : Sample
C1:24 : Sample
C1:25 : Sample
230
180
116
66
40
12
Ampka
pAmpka
H/R
H/R+Dex
H/R
H/R+Dex
GAPDH

Fig.6G

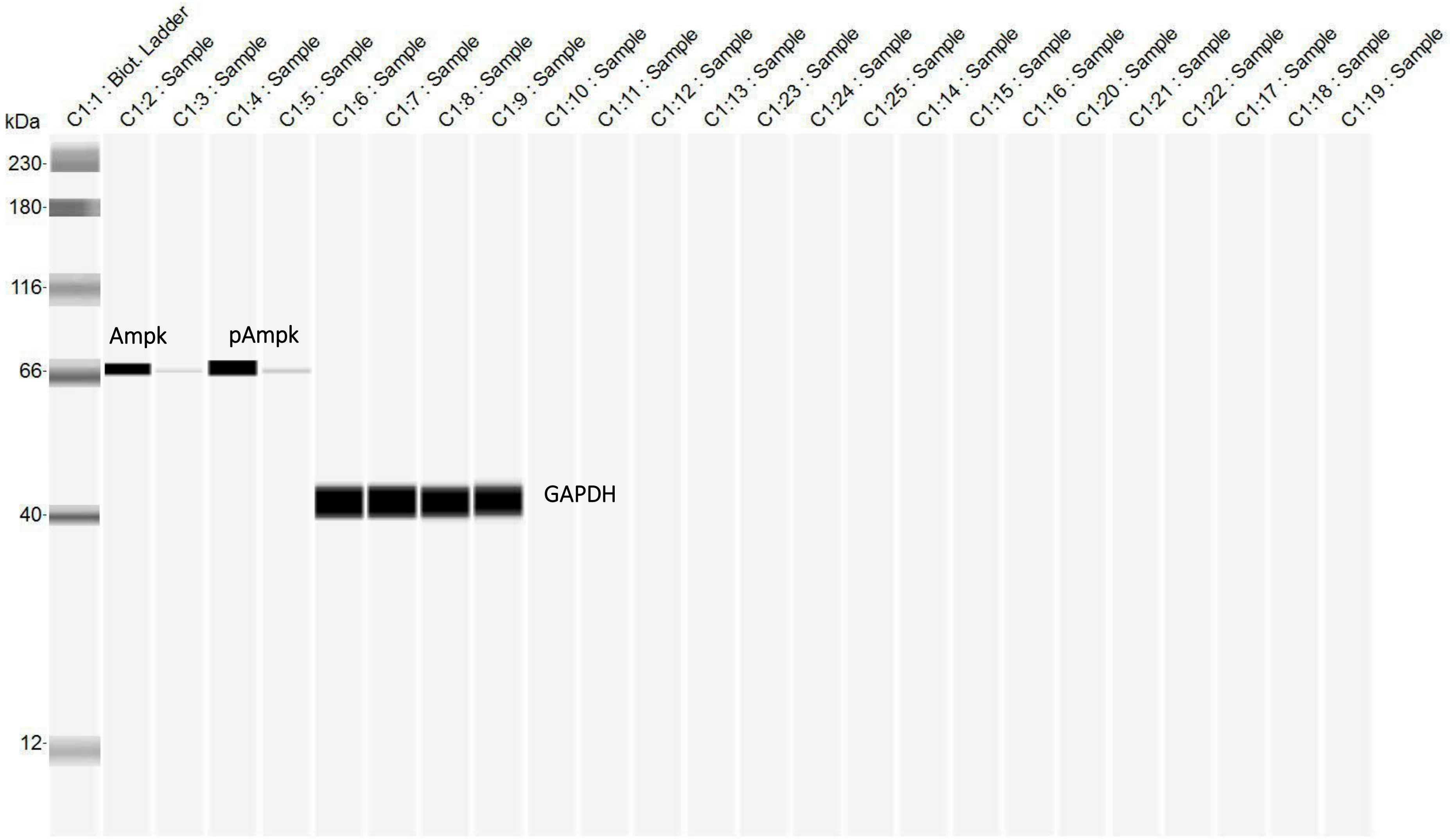
C1:1 : Biot. Ladder
C1:2 : Sample
C1:3 : Sample
C1:4 : Sample
C1:5 : Sample
C1:6 : Sample
C1:7 : Sample
C1:8 : Sample
C1:9 : Sample
C1:10 : Sample
C1:11 : Sample
C1:12 : Sample
C1:13 : Sample
C1:23 : Sample
C1:24 : Sample
C1:25 : Sample
C1:14 : Sample
C1:15 : Sample
C1:16 : Sample
C1:20 : Sample
C1:21 : Sample
C1:22 : Sample
C1:17 : Sample
C1:18 : Sample
C1:19 : Sample
kDa
230
180
116
66
40
12
Ampk
pAmpk
GAPDH

Fig.6I

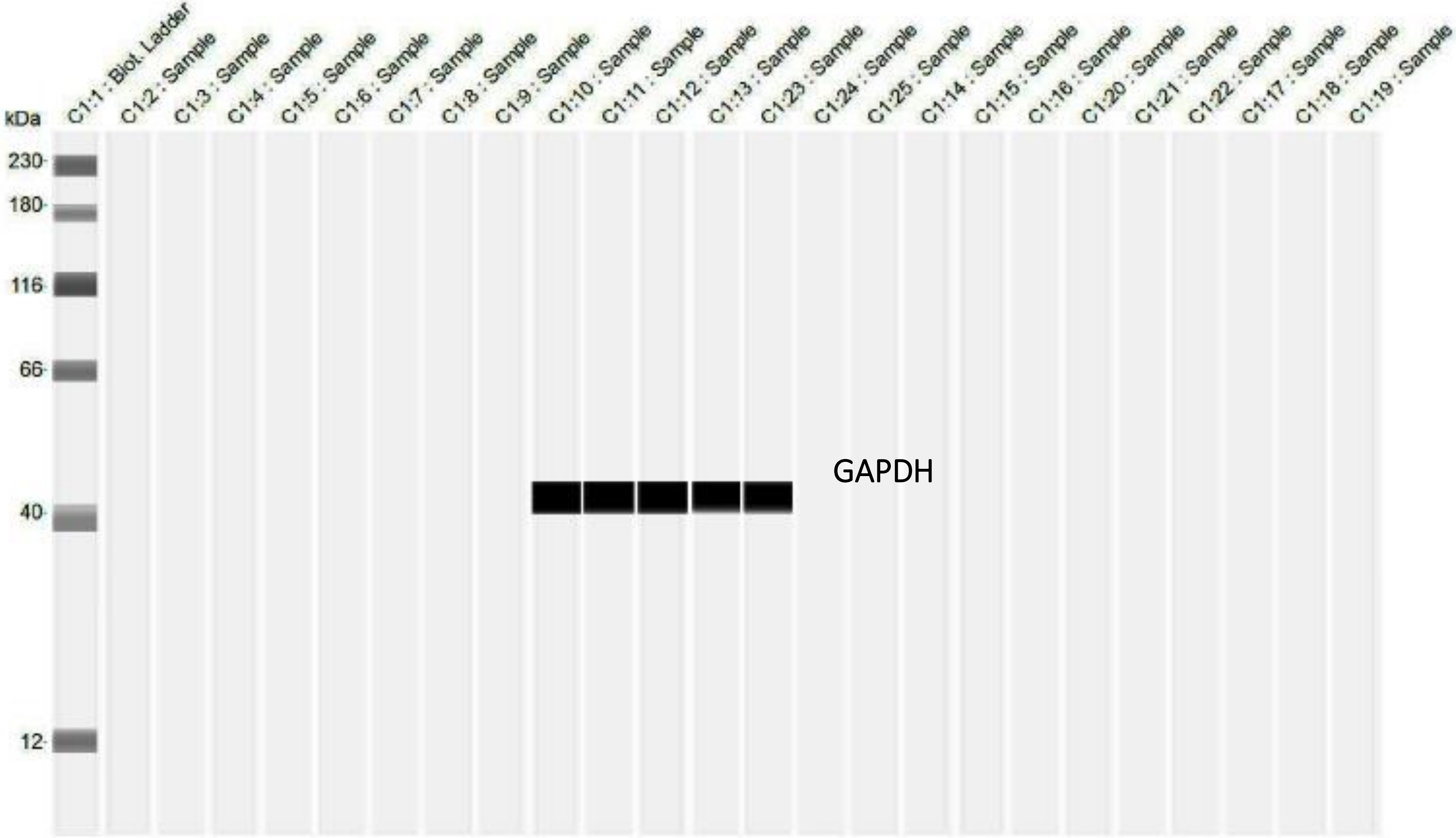
kDa
C1:1 : Biot. Ladder
C1:2 : Sample
C1:3 : Sample
C1:4 : Sample
C1:5 : Sample
C1:6 : Sample
C1:7 : Sample
C1:8 : Sample
C1:9 : Sample
C1:10 : Sample
C1:11 : Sample
C1:12 : Sample
C1:13 : Sample
C1:23 : Sample
C1:24 : Sample
C1:25 : Sample
C1:14 : Sample
C1:15 : Sample
C1:16 : Sample
C1:20 : Sample
C1:21 : Sample
C1:22 : Sample
C1:17 : Sample
C1:18 : Sample
C1:19 : Sample
230
180
116
66
40
12
GAPDH

Fig.6I

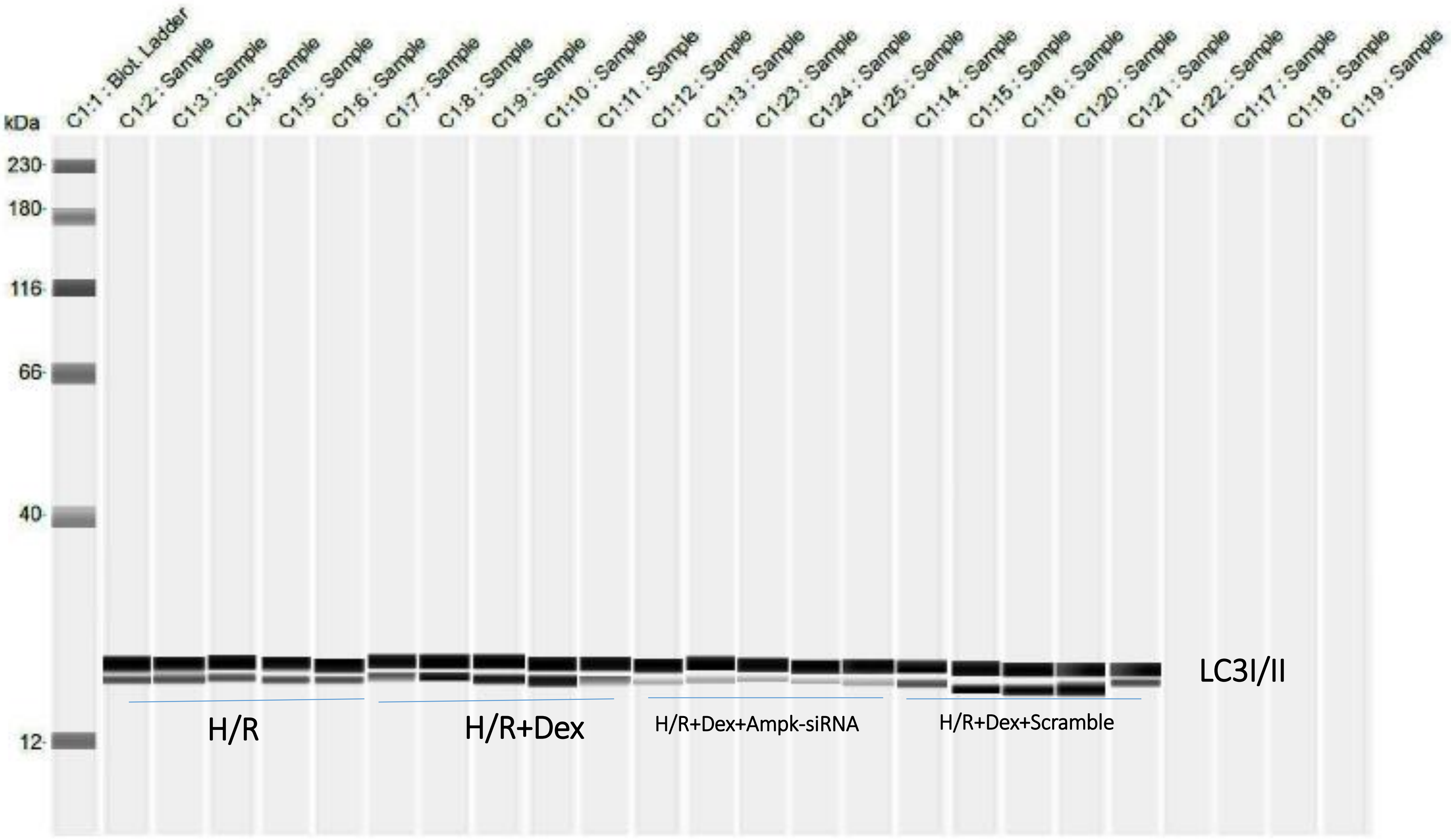
kDa
C1:1 : Biot. Ladder
C1:2 : Sample
C1:3 : Sample
C1:4 : Sample
C1:5 : Sample
C1:6 : Sample
C1:7 : Sample
C1:8 : Sample
C1:9 : Sample
C1:10 : Sample
C1:11 : Sample
C1:12 : Sample
C1:13 : Sample
C1:23 : Sample
C1:24 : Sample
C1:25 : Sample
C1:14 : Sample
C1:15 : Sample
C1:16 : Sample
C1:20 : Sample
C1:21 : Sample
C1:22 : Sample
C1:17 : Sample
C1:18 : Sample
C1:19 : Sample
230
180
116
66
40
12
LC3I/II
H/R
H/R+Dex
H/R+Dex+Ampk-siRNA
H/R+Dex+Scramble

Fig.6K

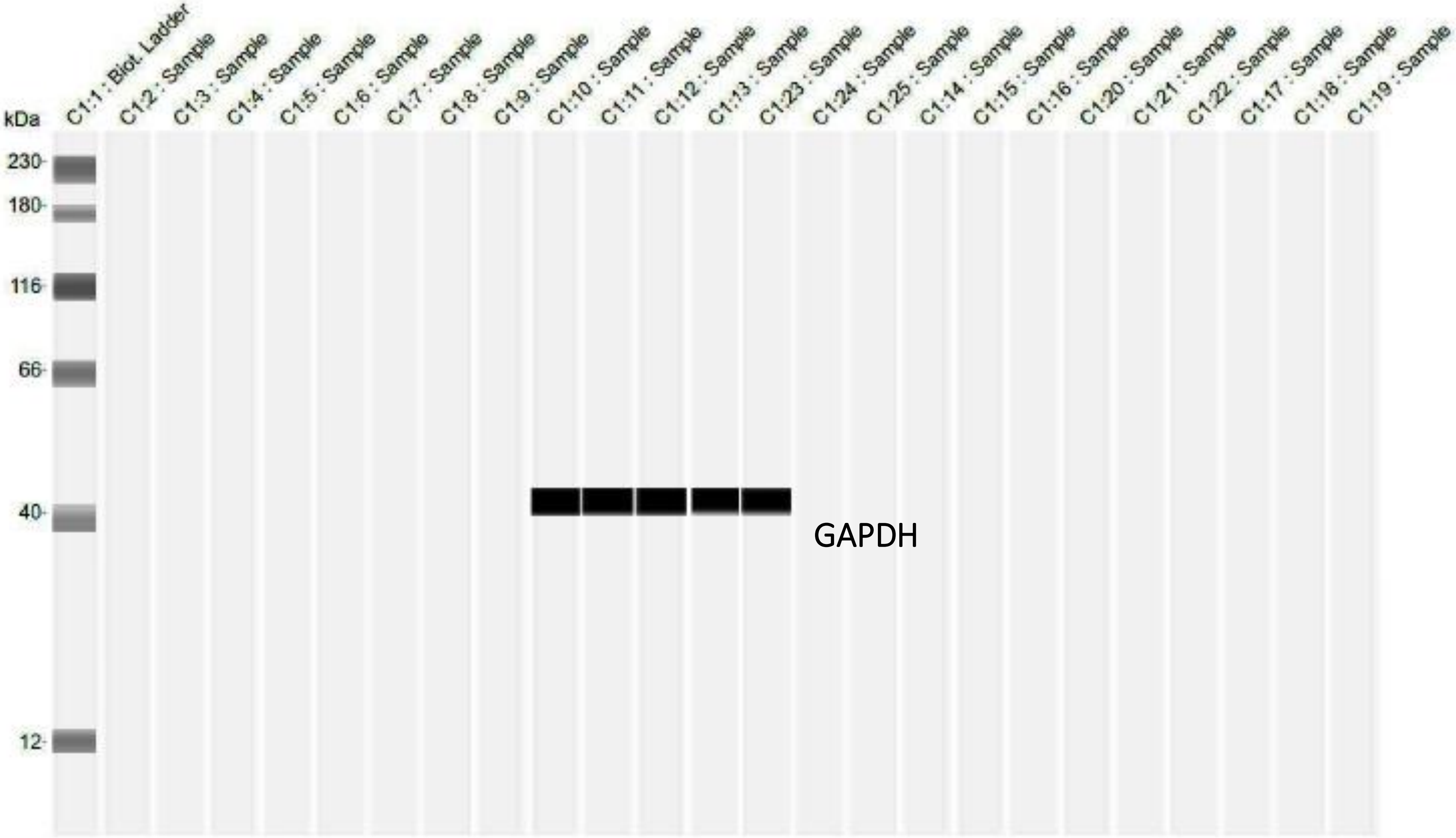
kDa
C1:1 : Biot. Ladder
C1:2 : Sample
C1:3 : Sample
C1:4 : Sample
C1:5 : Sample
C1:6 : Sample
C1:7 : Sample
C1:8 : Sample
C1:9 : Sample
C1:10 : Sample
C1:11 : Sample
C1:12 : Sample
C1:13 : Sample
C1:23 : Sample
C1:24 : Sample
C1:25 : Sample
C1:14 : Sample
C1:15 : Sample
C1:16 : Sample
C1:20 : Sample
C1:21 : Sample
C1:22 : Sample
C1:17 : Sample
C1:18 : Sample
C1:19 : Sample
230
180
116
66
40
12

GAPDH

Fig.6K

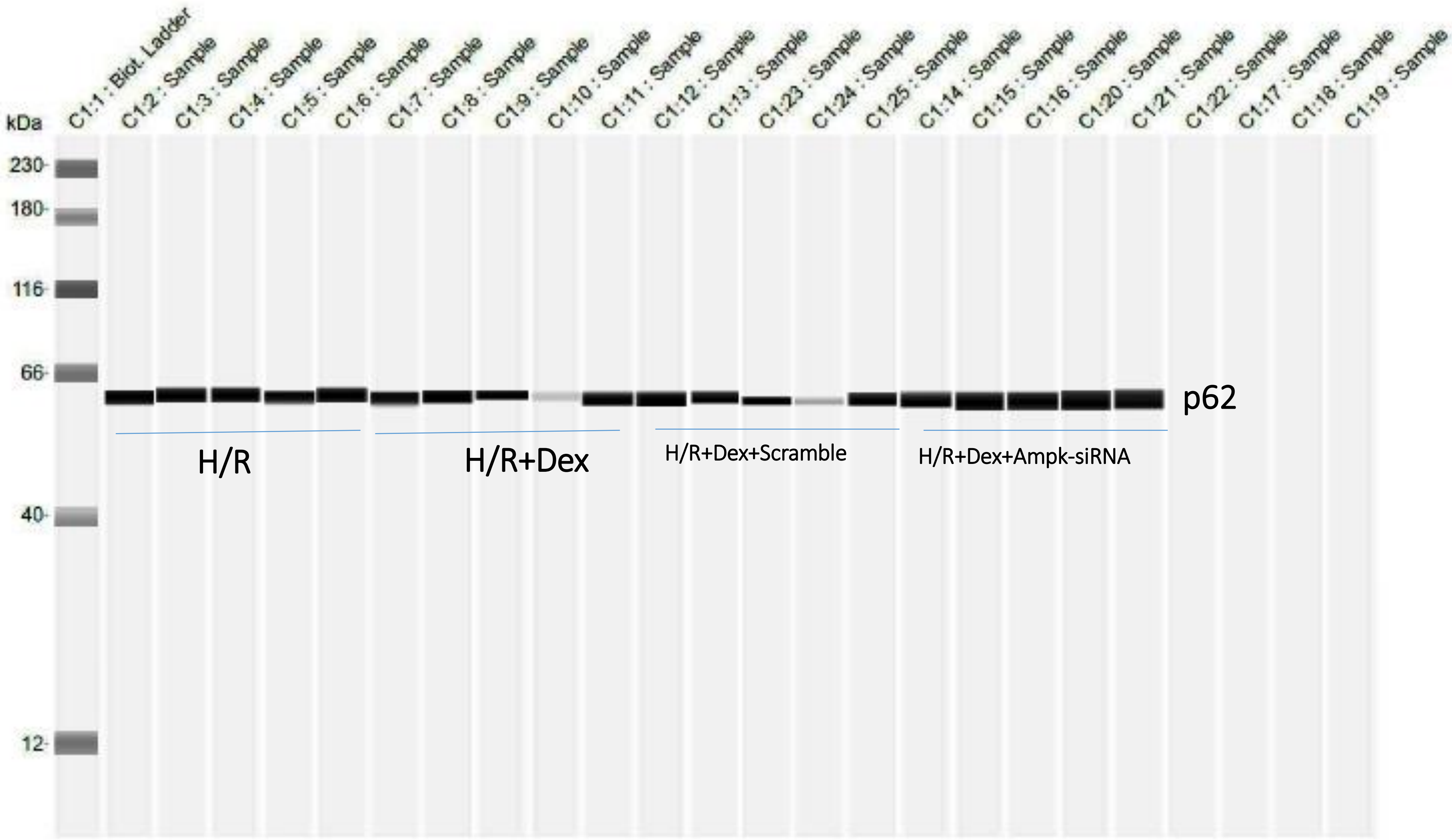

kDa
C1:1 : Biot. Ladder
C1:2 : Sample
C1:3 : Sample
C1:4 : Sample
C1:5 : Sample
C1:6 : Sample
C1:7 : Sample
C1:8 : Sample
C1:9 : Sample
C1:10 : Sample
C1:11 : Sample
C1:12 : Sample
C1:13 : Sample
C1:23 : Sample
C1:24 : Sample
C1:25 : Sample
C1:14 : Sample
C1:15 : Sample
C1:16 : Sample
C1:20 : Sample
C1:21 : Sample
C1:22 : Sample
C1:17 : Sample
C1:18 : Sample
C1:19 : Sample
230
180
116
66
40
12
p62
H/R
H/R+Dex
H/R+Dex+Scramble
H/R+Dex+Ampk-siRNA

Fig.7A

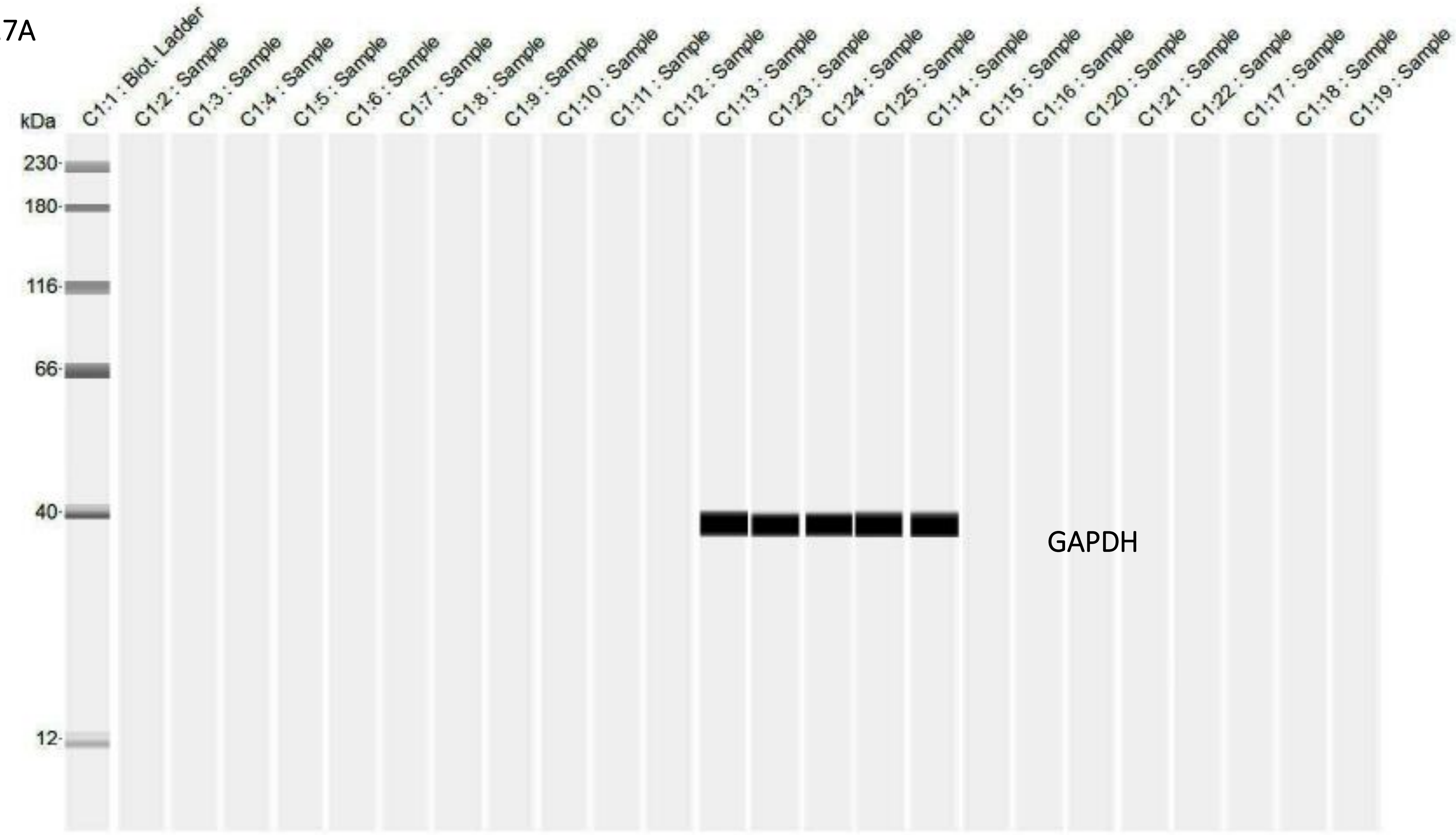

C1:1 : Biot. Ladder
C1:2 : Sample
C1:3 : Sample
C1:4 : Sample
C1:5 : Sample
C1:6 : Sample
C1:7 : Sample
C1:8 : Sample
C1:9 : Sample
C1:10 : Sample
C1:11 : Sample
C1:12 : Sample
C1:13 : Sample
C1:23 : Sample
C1:24 : Sample
C1:25 : Sample
C1:14 : Sample
C1:15 : Sample
C1:16 : Sample
C1:20 : Sample
C1:21 : Sample
C1:22 : Sample
C1:17 : Sample
C1:18 : Sample
C1:19 : Sample
kDa
230
180
116
66
40
12
GAPDH

Fig.7A

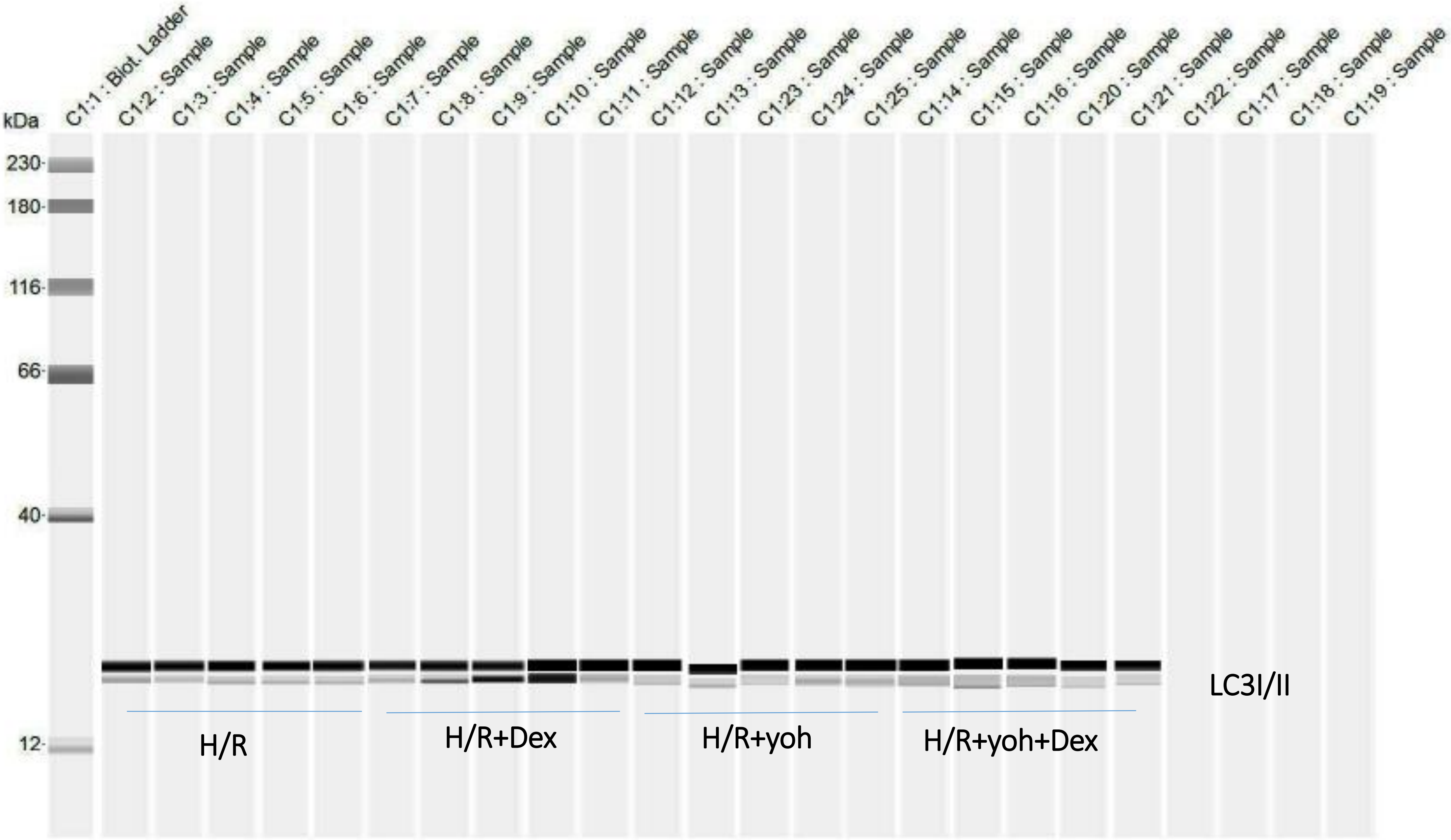

kDa
C1:1 : Biot. Ladder
C1:2 : Sample
C1:3 : Sample
C1:4 : Sample
C1:5 : Sample
C1:6 : Sample
C1:7 : Sample
C1:8 : Sample
C1:9 : Sample
C1:10 : Sample
C1:11 : Sample
C1:12 : Sample
C1:13 : Sample
C1:23 : Sample
C1:24 : Sample
C1:25 : Sample
C1:14 : Sample
C1:15 : Sample
C1:16 : Sample
C1:20 : Sample
C1:21 : Sample
C1:22 : Sample
C1:17 : Sample
C1:18 : Sample
C1:19 : Sample
230
180
116
66
40
12
LC3I/II
H/R
H/R+Dex
H/R+yoh
H/R+yoh+Dex

Fig.7C

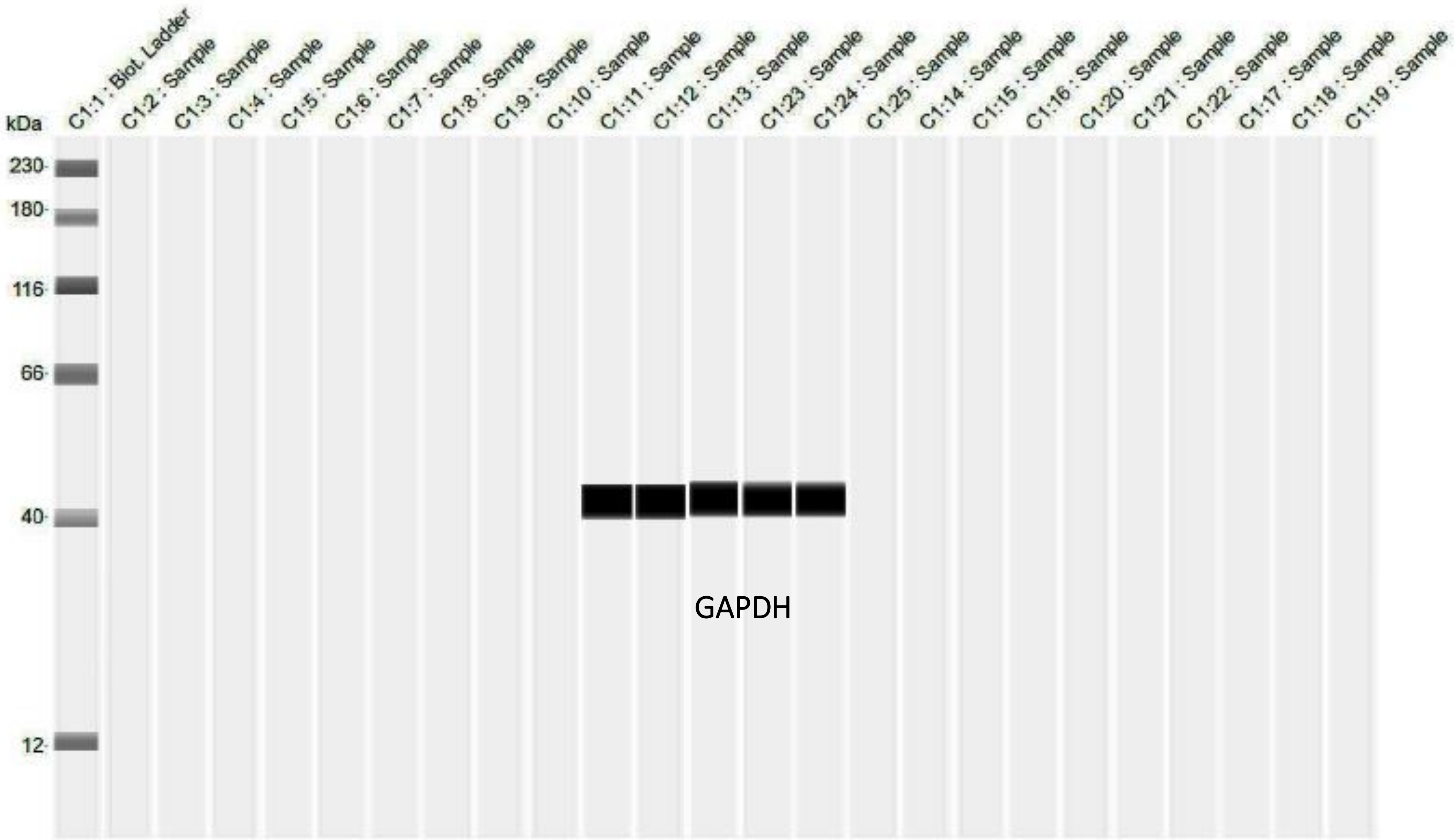

kDa
C1:1 : Biot. Ladder
C1:2 : Sample
C1:3 : Sample
C1:4 : Sample
C1:5 : Sample
C1:6 : Sample
C1:7 : Sample
C1:8 : Sample
C1:9 : Sample
C1:10 : Sample
C1:11 : Sample
C1:12 : Sample
C1:13 : Sample
C1:23 : Sample
C1:24 : Sample
C1:25 : Sample
C1:14 : Sample
C1:15 : Sample
C1:16 : Sample
C1:20 : Sample
C1:21 : Sample
C1:22 : Sample
C1:17 : Sample
C1:18 : Sample
C1:19 : Sample
230
180
116
66
40
12
GAPDH

Fig.7C

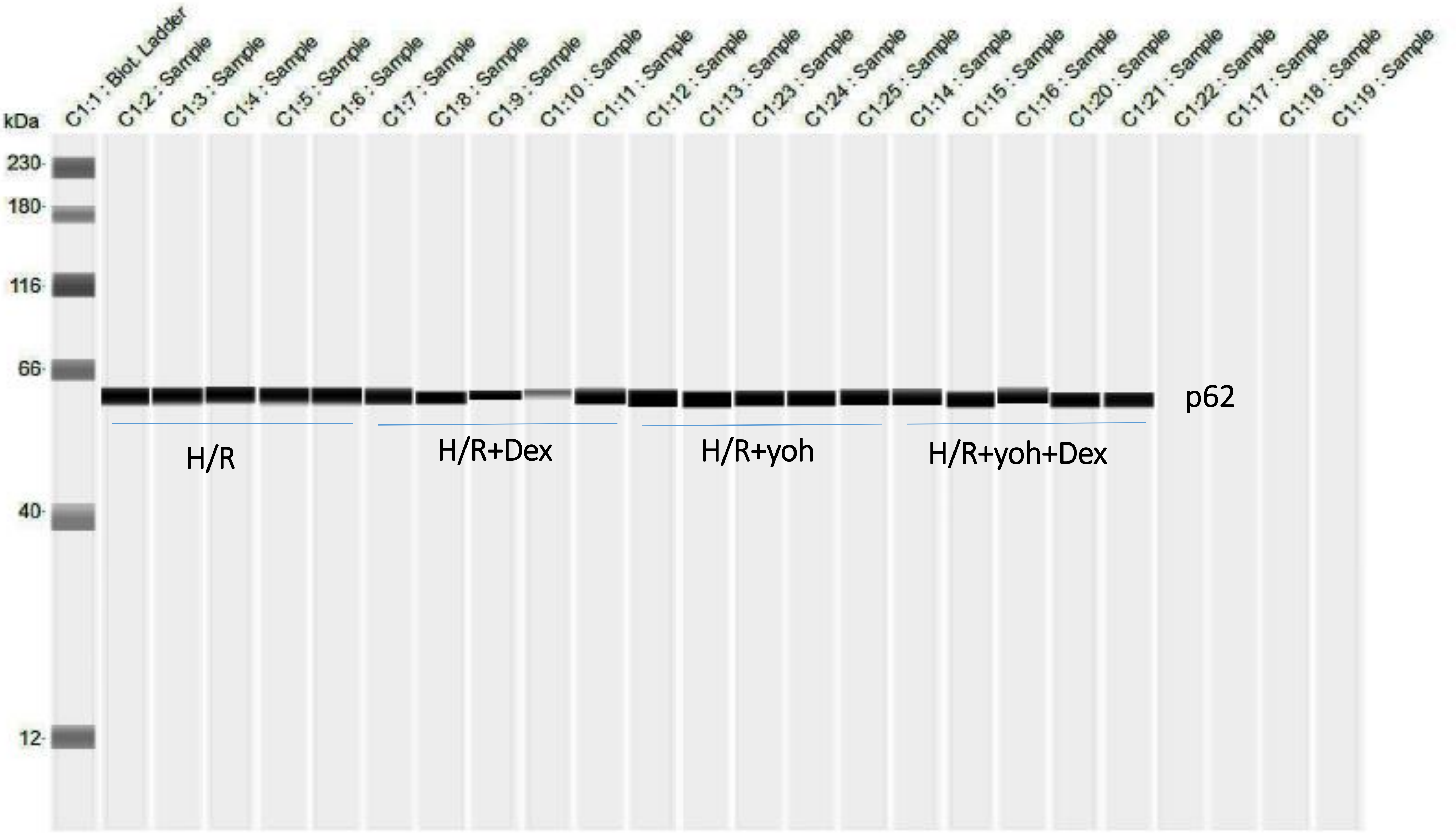
kDa
C1:1 : Biot. Ladder
C1:2 : Sample
C1:3 : Sample
C1:4 : Sample
C1:5 : Sample
C1:6 : Sample
C1:7 : Sample
C1:8 : Sample
C1:9 : Sample
C1:10 : Sample
C1:11 : Sample
C1:12 : Sample
C1:13 : Sample
C1:23 : Sample
C1:24 : Sample
C1:25 : Sample
C1:14 : Sample
C1:15 : Sample
C1:16 : Sample
C1:20 : Sample
C1:21 : Sample
C1:22 : Sample
C1:17 : Sample
C1:18 : Sample
C1:19 : Sample
230
180
116
66
40
12
p62
H/R
H/R+Dex
H/R+yoh
H/R+yoh+Dex

Fig.7G

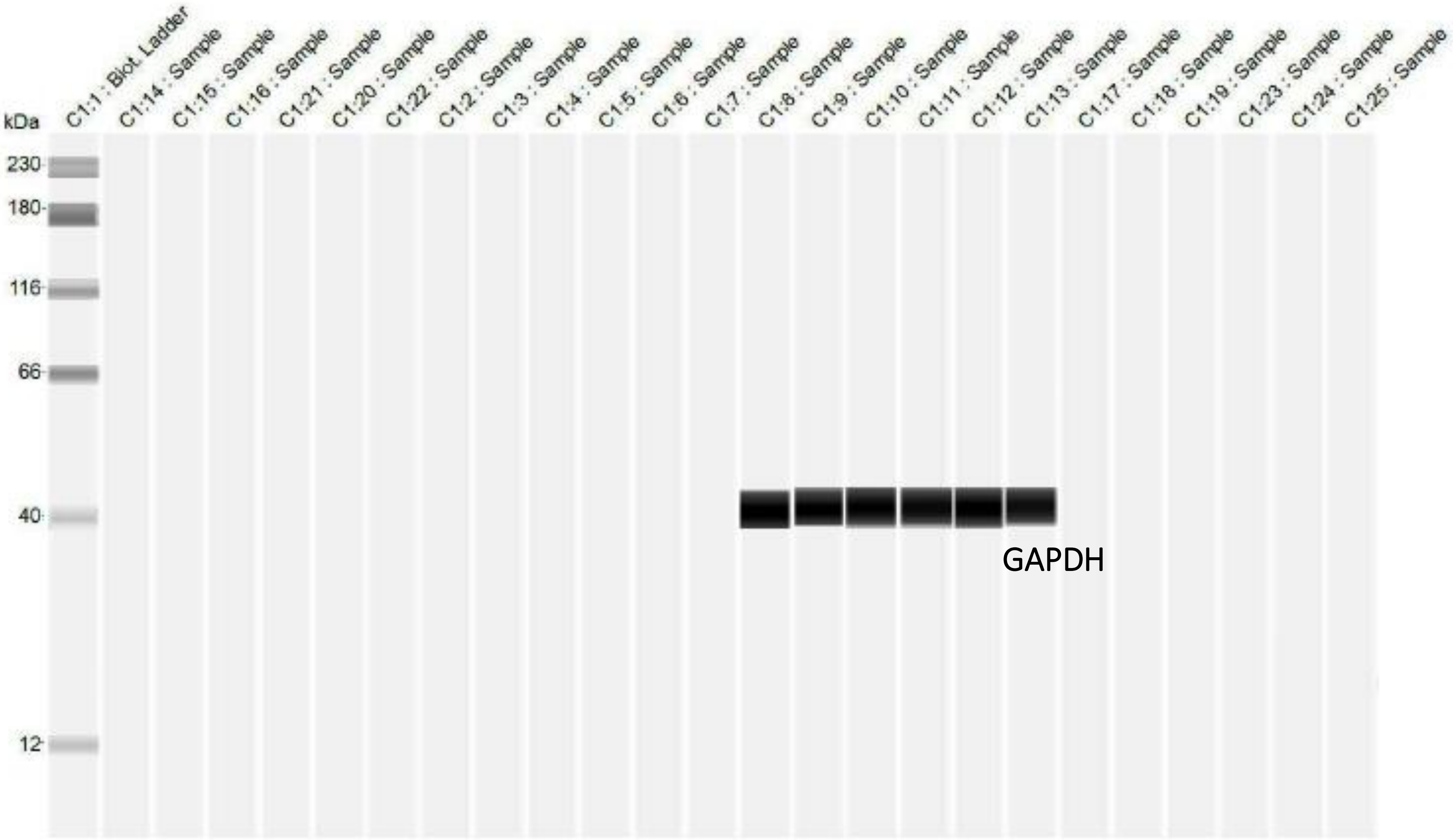
kDa
C1:1 : Biot. Ladder
C1:14 : Sample
C1:15 : Sample
C1:16 : Sample
C1:21 : Sample
C1:20 : Sample
C1:22 : Sample
C1:2 : Sample
C1:3 : Sample
C1:4 : Sample
C1:5 : Sample
C1:6 : Sample
C1:7 : Sample
C1:8 : Sample
C1:9 : Sample
C1:10 : Sample
C1:11 : Sample
C1:12 : Sample
C1:13 : Sample
C1:17 : Sample
C1:18 : Sample
C1:19 : Sample
C1:23 : Sample
C1:24 : Sample
C1:25 : Sample
230
180
116
66
40
12
GAPDH

Fig.7G

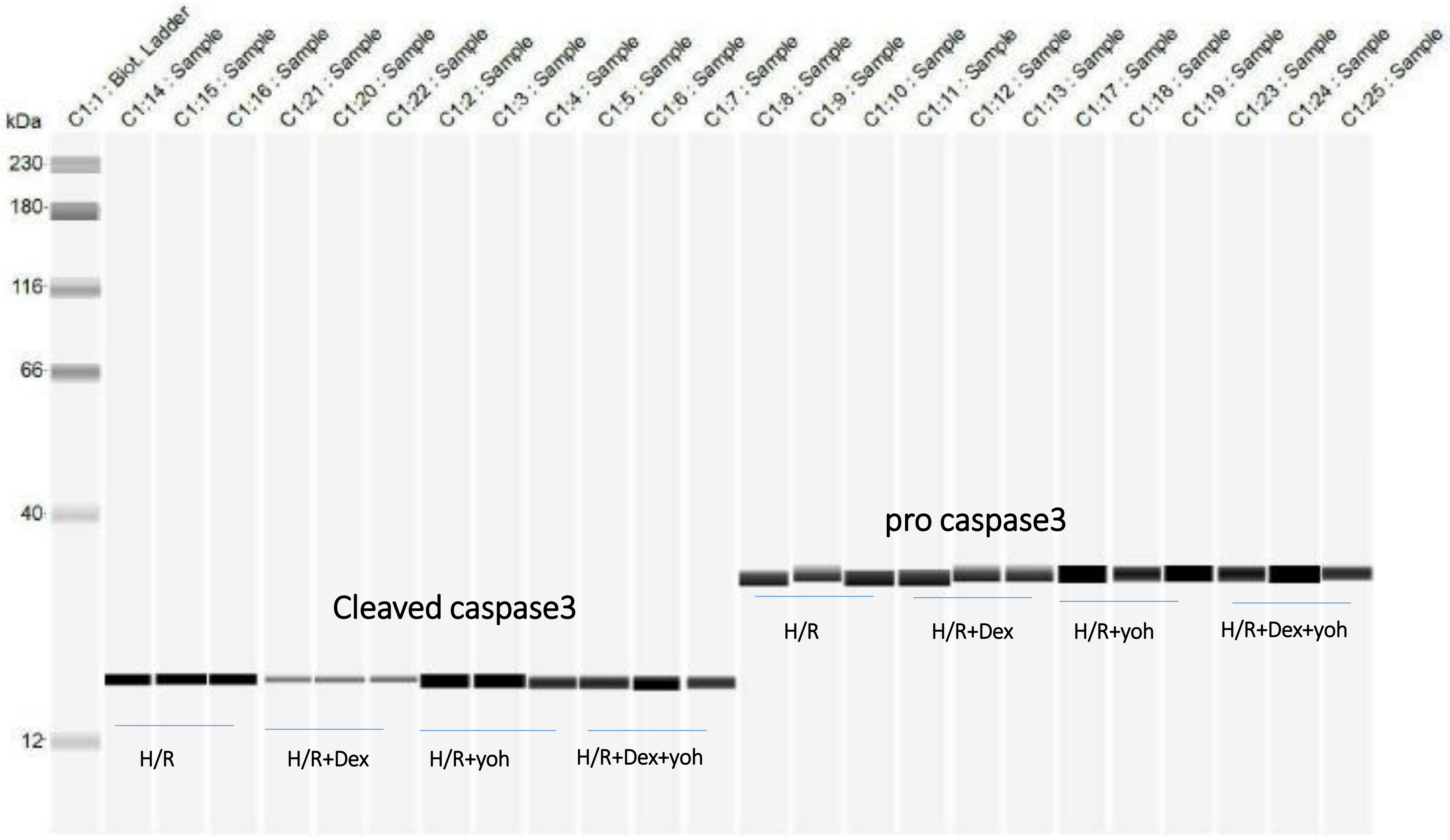
kDa
230
180
116
66
40
12
C1:1 : Biot. Ladder
C1:14 : Sample
C1:15 : Sample
C1:16 : Sample
C1:21 : Sample
C1:20 : Sample
C1:22 : Sample
C1:2 : Sample
C1:3 : Sample
C1:4 : Sample
C1:5 : Sample
C1:6 : Sample
C1:7 : Sample
C1:8 : Sample
C1:9 : Sample
C1:10 : Sample
C1:11 : Sample
C1:12 : Sample
C1:13 : Sample
C1:17 : Sample
C1:18 : Sample
C1:19 : Sample
C1:23 : Sample
C1:24 : Sample
C1:25 : Sample
pro caspase3
Cleaved caspase3
H/R
H/R+Dex
H/R+yoh
H/R+Dex+yoh
H/R
H/R+Dex
H/R+yoh
H/R+Dex+yoh

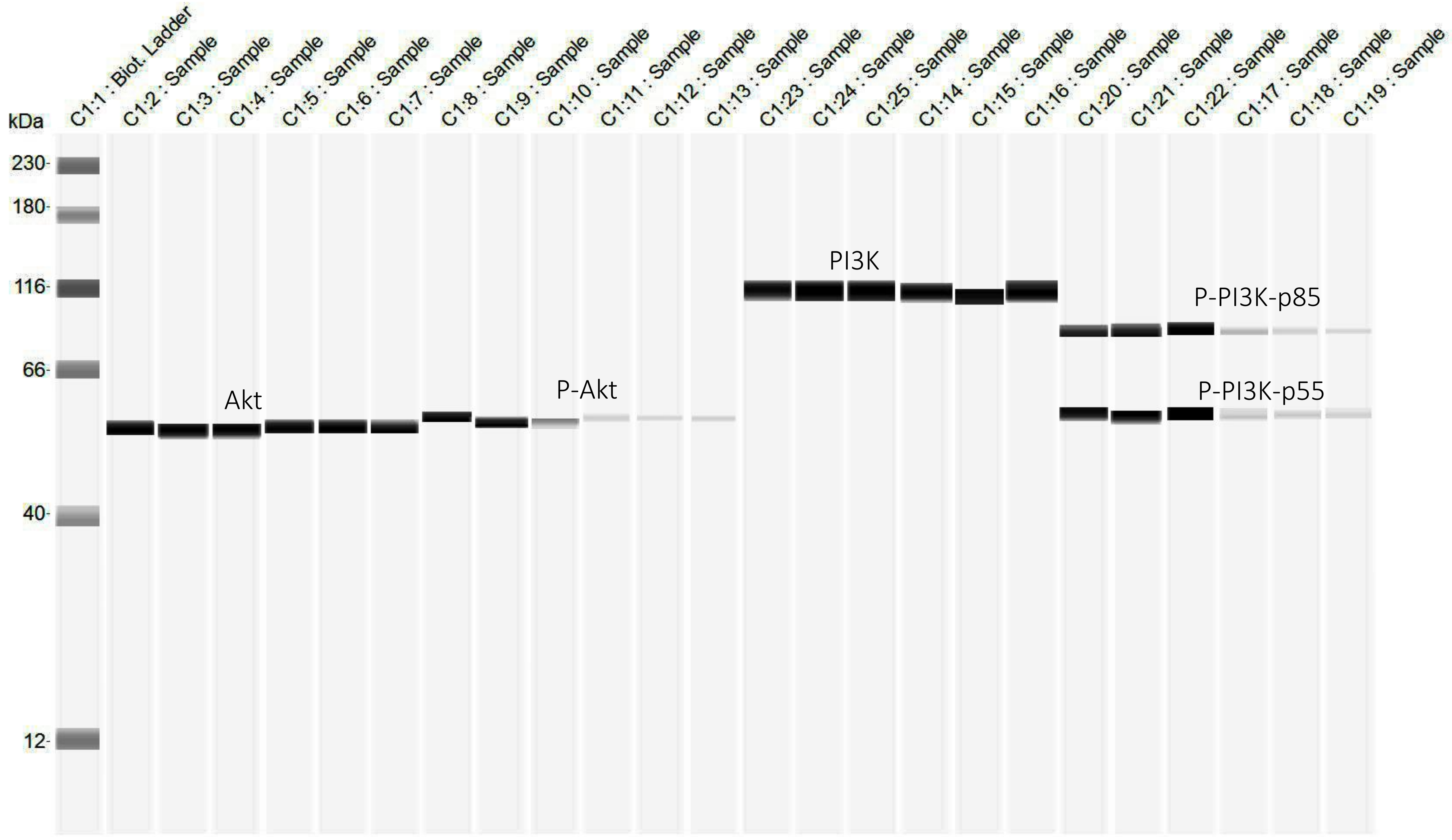
kDa
C1:1 : Biot. Ladder
C1:2 : Sample
C1:3 : Sample
C1:4 : Sample
C1:5 : Sample
C1:6 : Sample
C1:7 : Sample
C1:8 : Sample
C1:9 : Sample
C1:10 : Sample
C1:11 : Sample
C1:12 : Sample
C1:13 : Sample
C1:23 : Sample
C1:24 : Sample
C1:25 : Sample
C1:14 : Sample
C1:15 : Sample
C1:16 : Sample
C1:20 : Sample
C1:21 : Sample
C1:22 : Sample
C1:17 : Sample
C1:18 : Sample
C1:19 : Sample
230
180
116
66
40
12
Akt
P-Akt
PI3K
P-PI3K-p85
P-PI3K-p55

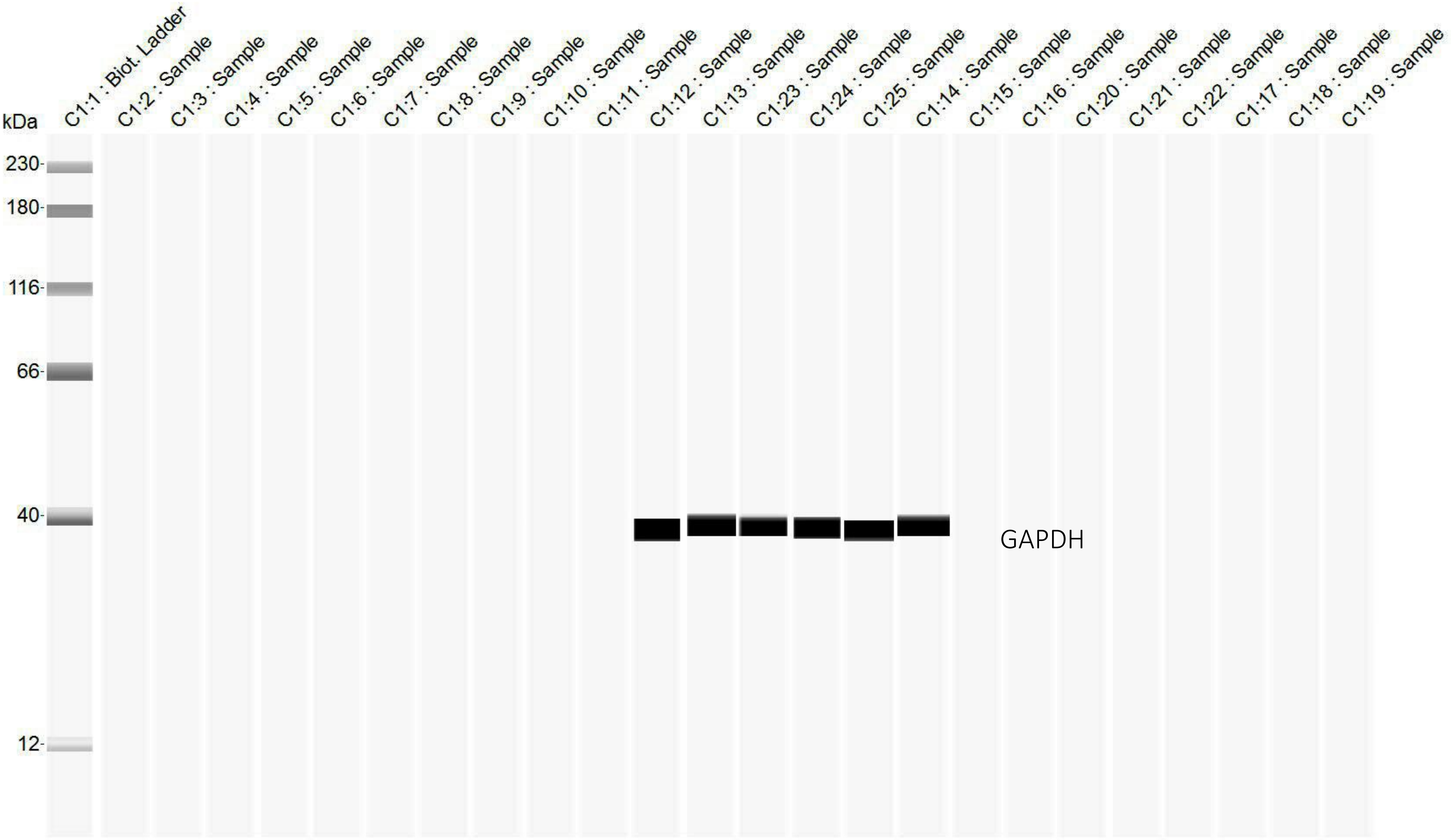

kDa
C1:1 : Biot. Ladder
C1:2 : Sample
C1:3 : Sample
C1:4 : Sample
C1:5 : Sample
C1:6 : Sample
C1:7 : Sample
C1:8 : Sample
C1:9 : Sample
C1:10 : Sample
C1:11 : Sample
C1:12 : Sample
C1:13 : Sample
C1:23 : Sample
C1:24 : Sample
C1:25 : Sample
C1:14 : Sample
C1:15 : Sample
C1:16 : Sample
C1:20 : Sample
C1:21 : Sample
C1:22 : Sample
C1:17 : Sample
C1:18 : Sample
C1:19 : Sample
230-
180-
116-
66-
40-
12-
GAPDH
