# Supplementary material for: Dexmedetomidine Protects Human Cardiomyocytes Against Ischemia-Reperfusion Injury Through α2-Adrenergic Receptor/AMPK-Dependent Autophagy
Source: Front Pharmacol. 2021 May 21;12:615424. doi: 10.3389/fphar.2021.615424 (PMC8176440; doi:10.3389/fphar.2021.615424)
Supplement: Supplementary file 4 [file DataSheet2.docx]

Supplemental table S1 Reagents

| Name | Company | Catalog No. |
| --- | --- | --- |
| Dexmedetomidine(Dex) | Sigma-Aldrich | 1179333 |
| Bafilomycin A1 | Sigma-Aldrich | 19148 |
| DMEM/F12 | Gibco | A4192001 |
| TUNEL Kit | Beyotime | C1086 |
| WGA | Invitrogen | W11261 |
| Triton X-100 | Sigma-Aldrich | T9284 |
| HBSS | ThermoFisher Scientific, Pittsburgh, PA, USA | 14175103 |
| BCA kit | ThermoFisher Scientific | A53227 |
| human iPSC line | Allele Biotechnology, San Diego, California, USA | del-AR1034ZIMA 001 |
| STEMdiff Cardiomyocyte Differentiation Kit | STEMCELL Technologies, Vancouver, British Columbia, Canada | 5010 |
| paraformaldehyde(PFA) | Sigma-Aldrich | 158127 |
| 4',6-diamidino-2-phenylindole(DAPI) | ThermoFisher Scientific | D3571 |
| Wes module | proteinsimple | 12-230kD |
| RIPA lysis buffer | Beyotime, Shanghai, China | P0013B |
| yohimbine | Sigma-Aldrich | 731241 |
| AMPK siRNA | Santa Cruz Biotechnology | Sc-29673 |

| Supplemental Table S2 Antibodies | |  |
| --- | --- | --- |
| Name | Company | Catalog No. |
| cardiac troponin T | Abcam | ab8295 |
| sarcomeric α-actinin | Abcam | ab9465 |
| LC3 | Cell Signaling | 4108 |
| AMPKα1/2 | Cell Signaling | 2532 |
| p-AMPK(T172) | Cell Signaling | 2535 |
| P62 | Cell Signaling | 5114 |
| Akt | Abcam | ab8805 |
| pAkt | Abcam | ab8933 |
| PI3K | Cell Signaling | 4249 |
| pPI3K | Cell Signaling | 4228 |
| GAPDH | Abcam | ab8245 |
